# Dynamic trajectories of the triglyceride-glucose index link to all-cause hospital mortality in patients with hypertension and kidney failure: a multicenter study

**DOI:** 10.3389/fendo.2025.1698418

**Published:** 2026-01-09

**Authors:** Yanqun Huang, Xin Gan, Hui Liang, Senhu Tang, Junfan Chen, Yinglong Shi

**Affiliations:** 1 Department of Medical Equipment, the First Affiliated Hospital of Guangxi Medical University, Nanning, Guangxi, China; 2 Department of Neurosurgery, the First Affiliated Hospital of Guangxi Medical University, Nanning, Guangxi, China; 3 Department of Cardiology, Liuzhou People’s Hospital, Affiliated of Guangxi Medical University, Liuzhou, Guangxi, China; 4 Department of Nephrology, the First Affiliated Hospital of Guangxi Medical University, Nanning, Guangxi, China

**Keywords:** hypertension, kidney failure, mortality, time-weighted average TyG, triglyceride-glucose (TyG) index, TyG trajectories

## Abstract

**Background:**

Chronic kidney disease and hypertension form a vicious cardiorenal cycle, exacerbating metabolic dysfunction and mortality. The triglyceride-glucose (TyG) index, a surrogate for insulin resistance, has shown prognostic value in cardiovascular and renal diseases. Previous research analyzed single-timepoint TyG, ignoring longitudinal trajectories during hospitalization. We aimed to investigate TyG trajectories and their association with hospital mortality in patients with hypertension and kidney failure (KF).

**Methods:**

Patients diagnosed with hypertension and KF were retrospectively retrieved from MIMIC-IV and a private dataset. Patients were clustered into four TyG trajectory groups using K-means clustering. A novel time-weighted average TyG (WATyG) metric was developed to quantify cumulative metabolic exposure. Logistic regression, restricted cubic spline (RCS) models, and subgroup analyses examined associations between TyG dynamics and mortality.

**Results:**

A total of 2,038 patients from MIMIC-IV and 1,266 from a private dataset were analyzed, with mortality rates of 28.41% and 7.03%, respectively. Four TyG trajectories were identified: rapidly increasing (Cluster 1), rapidly decreasing (Cluster 2), persistent high (Cluster 3), and stable low (Cluster 4). Clusters 1 and 3 had significantly higher mortality rates than Clusters 2 and 4 (all P<0.001). In MIMIC-IV, mortality rates were 38.8%/35.0% for Clusters 1/3 versus 22.6%/22.7% for Clusters 2/4, while the private dataset showed rates of 18.5%/7.6% (Clusters 1/3) versus 5.5%/4.0% (Clusters 2/4). Using Cluster 1 as reference in the adjusted model, Cluster 2 (OR 0.546, P=0.007) and Cluster 4 (OR 0.492, P<0.001) showed lower mortality risks in MIMIC-IV, with consistent trends in the private dataset. WATyG was linearly associated with an increased risk of mortality (OR 1.505, P<0.001 in MIMIC-IV).

**Conclusions:**

Dynamic TyG trajectories are linked to mortality risk in patients with hypertension and KF. WATyG improves risk stratification via cumulative metabolic exposure. Longitudinal TyG monitoring holds potential value for optimizing clinical decision-making by enabling continuous assessment of metabolic risk.

## 1 Introduction

Chronic kidney disease (CKD) is a global health challenge affecting over 850 million individuals worldwide (1, 2), with 1.2 million deaths attributed to the condition in 2017, and projections indicate this number will rise to 2.2 million by 2040 under best-case scenarios and potentially reach 4.0 million annually in worst-case scenarios (3). Hypertension, a key risk factor for cardiovascular disease and kidney failure (KF), coexists in about 50% of CKD patients (4, 5). Hypertension and impaired kidney function form a bidirectional relationship. Hypertension accelerates renal decline, while impaired kidney function exacerbates blood pressure elevation. This creates a vicious cycle that synergistically increases cardiovascular risk and all-cause mortality, especially in advanced CKD stages (4, 6). Among patients with hypertension and KF, diabetic kidney disease (DKD) and cardio-renal-metabolic syndrome (CRMS) are common comorbidities that exacerbate metabolic dysregulation and adverse outcomes (7, 8). Despite therapeutic advances, hospital mortality remains high, underscoring the urgent need for novel prognostic markers to improve risk stratification in this vulnerable population (1, 4).

Insulin resistance (IR), a hallmark of metabolic syndrome and a driver of cardiorenal syndromes, is a focus of our study. The triglyceride - glucose (TyG) index, derived from fasting triglyceride (TG) and fasting blood glucose (FBG) levels, is a practical surrogate for IR. It has advantages in populations with metabolic disorders and its prognostic value has been explored in various clinical contexts (9, 10). High TyG levels are linked to adverse cardiovascular and renal outcomes, such as arterial stiffness, heart failure, and CKD progression (9–16). Notably, hypertriglyceridemia, a core component of TyG index calculation, involves complex molecular pathways including the dysregulation of lipoprotein lipase (LPL) activity and apolipoprotein (Apo) C-II/C-III/A-V function, and angiopoietin-like proteins (ANGPTL3/4/8) mediation, which collectively impair triglyceride clearance and promote atherogenic lipoprotein accumulation (17–20). Molecular perturbations link insulin resistance and cardiorenal damage, reinforcing the TyG index as a biologically plausible prognostic marker in hypertensive KF patients.

Recent studies have highlighted the TyG index’s relevance in high-risk populations. Among critically ill patients with hypertension, elevated TyG levels correlated with increased acute kidney injury (AKI) incidence and all-cause mortality (15). Similarly, in patients with heart diseases, higher TyG indices predicted worse outcomes, including prolonged hospitalization and increased mortality (15, 21, 22). Systematic reviews confirm the TyG index’s link to hypertension and its predictive capacity for major adverse cardiovascular events in hypertensive patients (23, 24). However, most studies relied on single time-point TyG measurements, overlooking longitudinal trajectories in hypertensive KF patients at high risk of cardiorenal metabolic deterioration (25, 26). While TyG has been validated as a static predictor, the temporal link between its dynamic changes and clinical outcomes remains unclear. Additionally, prior studies often overlooked patients’ pharmacological backgrounds, such as hypoglycemic, lipid-modifying and antihypertensive medications. As these agents directly regulate triglyceride metabolism, insulin sensitivity and cardiorenal function, factors that may confound the TyG index-mortality association, medication adjustment in multivariate analyses is essential to validate the independent prognostic value of TyG trajectories.

Against this background, the present study aimed to investigate the dynamic trajectory of the TyG index and its association with all-cause hospital mortality in patients with hypertension and KF. We used longitudinal hospitalization data to analyze how TyG fluctuations reflect metabolic dysregulation and impact clinical outcomes in this high-risk population. We comprehensively accounted for medication use and key comorbidities such as DKD, CRMS and other relevant conditions to ensure a robust assessment of the independent association between TyG trajectories and mortality. These findings may offer actionable insights for metabolic risk stratification in this vulnerable group against the background of the global burden of cardio-renal diseases.

## 2 Methods and materials

### 2.1 Study population

We conducted a multicenter, observational, retrospective study to evaluate the association of the TyG trajectories with mortality in patients with hypertension and KF, utilizing records from the Medical Information Mart for Intensive Care IV (MIMIC-IV) database (version 3.1) and a private dataset in a tertiary hospital in China. The MIMIC-IV 3.1 database (https://mimic.mit.edu) is a publicly accessible critical care repository maintained by the Massachusetts Institute of Technology, containing records from over 220,000 patients, including demographics, laboratory measurements, medications, and diagnoses classified according to International Classification of Diseases (ICD)-9 and ICD-10 codes. One author (Yanqun Huang) obtained Institutional Review Board approval (certification number: 57439457) to access the database. The private dataset contains 136,051 patients with demographics, laboratory measurements, medications, and diagnoses identified by ICD-10 codes. In the private dataset, personal information was anonymized prior to remote data access, ensuring the data were used in an anonymous and safe manner. And this study was approved by the Medical Ethics Committee of the First Affiliated Hospital of Guangxi Medical University (approval number 2025-E0712).

**Figure 1:**
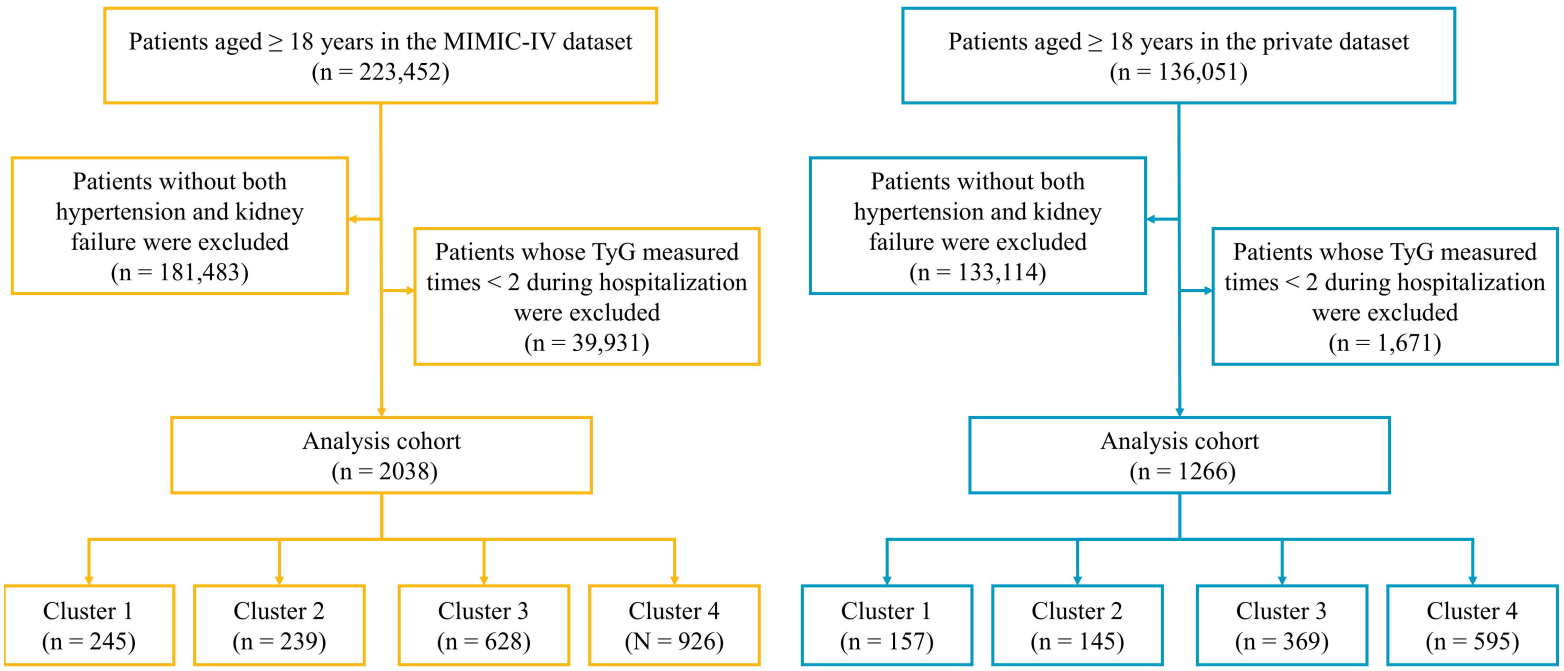
Flowchart of the study population.

Patients with hypertension and KF were identified using ICD-9 codes (hypertension: 401-405; KF: 583-586) and ICD-10 codes (hypertension: I10-I16; KF: N17-N19). From 223,452 patients in MIMIC-IV and 136,051 in the private dataset, we excluded 181,483 (MIMIC-IV) and 133,114 (private) patients who did not have both hypertension and KF, and 39,931 (MIMIC-IV) and 1,671 (private) patients with<2 TyG measurements during hospitalization. The final cohort comprised 2,038 patients in MIMIC-IV and 1,266 in the private dataset (**Figure 1**).

### 2.2 Assessment of the TyG trajectories and WATyG

Time-varying TG and FBG values recorded daily for each patient during hospitalization were retained. When multiple measurements of the same indicator were obtained on a single day, the daily mean was calculated to represent intra-day values. The TyG index was calculated as ln(TG [mg/dL] × FBG [mg/dL]/2). All daily available TyG measurements were retained. For each patient, the TyG trajectory was defined by changes between the first and last TyG measurements.

Patients often exhibited irregular TyG measurement patterns and uneven time intervals between measurements during hospitalization. Direct comparison of cumulative TyG (cumTyG) values, which was calculated as the sum of trapezoidal areas between consecutive measurements (27), may introduce bias, as patients with longer hospital stays or more frequent measurements inherently accumulate higher cumTyG values irrespective of actual TyG dynamics. To better characterize individual TyG changes, we introduced a novel metric: the time-weighted average TyG (WATyG). Inspired by methodologies for cumTyG (27) and time-weighted average glucose (28), WATyG accounts for variations in measurement frequency and intervals to some degree. This metric overcomes the limitation of comparing raw cumTyG values across patients with heterogeneous measurement patterns. Specifically, WATyG was derived by normalizing cumTyG by the time span between the first and last measurements, ensuring comparability across patients with varying hospitalization durations. The formula was defined as:


$$WATyG\;=\left(\overset{n-1}{\underset{i=1}{\sum }}(\frac{TyG_{i}+\;TyG_{i+1})}{2}*\left(D_{i+1}-\;D_{i}\right)\right)/\left(D_{n}-\;D_{1}\right)$$

Where *n* was the total number of TyG measurements for a patient, *TyG_i_* was the TyG value at the *i*-th measurement, *D_i_* was the time point (e.g., days after admission) of the *i*-th measurement.

### 2.3 Covariates

For each dataset, baseline clinical characteristics recorded within the first 48 hours after admission were extracted, including demographics (age and gender), hospitalization details (length of stay and discharge outcome), 14 comorbidities, 4 types of drugs, and 18 laboratory indicators with ≤30% missing values. The 14 comorbidities included diabetic kidney disease (DKD), cardio-renal-metabolic syndrome (CRMS), hyperlipidemia, heart failure, ischaemic heart disease, arrhythmia, stroke, peripheral vascular disease (PVD), diabetes, respiratory failure, cancer, anemia, electrolyte disturbance and chronic obstructive pulmonary disease (COPD). The 4 types of drugs included anti-hypertensive drugs (angiotensin converting enzyme inhibitors/angiotensin II receptor blockers [ACEI/ARB], loop diuretics, beta blockers, calcium channel blockers), anti-platelet drugs (aspirin), hypoglycemic drugs (insulin, oral hypoglycemic drugs) and lipid-modifying drugs (statins). The 18 laboratory indicators included TG, FBG, estimated glomerular filtration rate (eGFR), red blood cell (RBC), white blood cell (WBC), haemoglobin, red blood cell distribution width (RDW), haematocrit, platelet, creatinine, serum calcium (Ca), serum chloride (Cl), serum sodium (Na), serum potassium (K), alanine aminotransferase (ALT), alkaline phosphatase (ALP), aspartate aminotransferase (AST) and total bilirubin (BIL). Missing baseline laboratory indicators were imputed using the Random Forest algorithm, utilizing all non-missing variables as predictors in the imputation model.

### 2.4 Statistical analysis

Patients were first divided into four TyG trajectory clusters based on TyG change patterns using K-means clustering analysis and the elbow method (29). Continuous variables with normal distributions were presented as mean ± standard deviation (SD), while non-normally distributed variables were expressed as median (interquartile range, IQR). Categorical variables were reported as frequencies (percentages). Group comparisons employed χ² tests for categorical variables, ANOVA for normally distributed continuous variables, and Kruskal-Wallis tests for non-parametric continuous variables.

Given the limitations of cumulative TyG (cumTyG) in accounting for measurement heterogeneity, we focused on time-weighted average TyG (WATyG) as the primary exposure metric. WATyG was analyzed both continuously and categorically (quartiles: Q1 [low], Q2 [lower-middle], Q3 [upper-middle], Q4 [high]). Logistic regression models examined relationships between WATyG (continuous and quartiles) and mortality, reporting odds ratios (ORs) with 95% confidence intervals (CIs). Univariate analysis identified clinically relevant covariates. Four models were constructed: Model 1 (unadjusted), Model 2 (adjusted for age and gender), Model 3 (adjusted for demographics, 14 comorbidities and 18 baseline laboratory indicators), and Model 4 (adjusted for demographics, 14 comorbidities, 18 baseline laboratory indicators and 4 types of drugs). Restricted cubic spline (RCS) regression with three knots explored potential non-linear associations between WATyG and mortality across the four trajectory clusters.

Subgroup analyses and interaction tests evaluated consistency of WATyG-mortality associations across key strata: age (≤65 vs. >65 years) and gender; with or without DKD,CRMS, hyperlipidemia, heart failure, ischaemic heart diseases, arrhythmia, stroke, PVD, diabetes, respiratory failure, cancer, anemia, electrolyte disturbance, COPD; and presence or absence of use of anti-hypertensive drugs, anti-platelet drugs, hypoglycemic drugs, and lipid-modifying drugs. Likelihood ratio tests assessed interactions between WATyG and stratification variables. Statistical significance was defined as two-sided P< 0.05.

## 3 Results

### 3.1 Demographic and clinical characteristics

**Figure 2:**
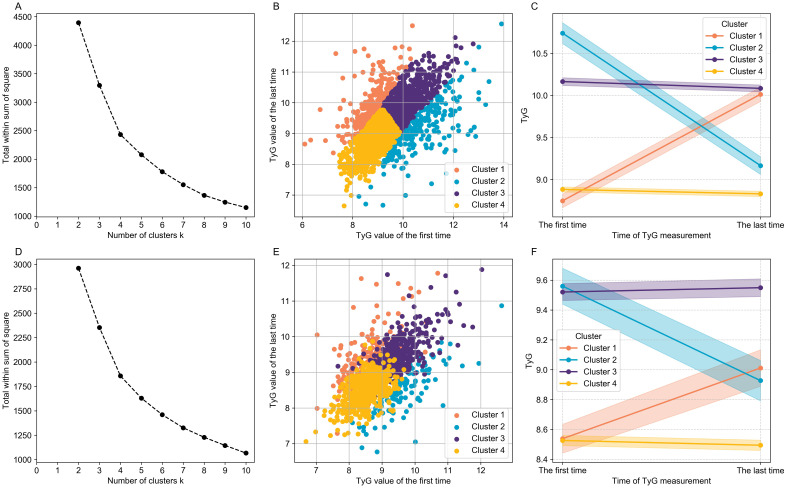
Clusters of the TyG change trajectory in patients with hypertension and kidney failure during hospitalization in MIMIC-IV **(A–C)** and the private dataset **(D–F)**. Cluster 1 (Orange): Rapidly Increasing Group; Cluster 2 (Blue): Rapidly Decreasing Group; Cluster 3 (Purple): Persistent High Group; Cluster 4 (Yellow): Stable Low Group.

In both datasets, patients were clustered into four distinct groups based on TyG change trajectories during hospitalization (**Figure 2**). **Tables 1** and **2** present baseline characteristics across these clusters in MIMIC-IV and the private dataset, respectively. In MIMIC-IV (**Table 1**, **Figures 2A-C**), Cluster 1 (rapidly increasing group, n=245) showed a marked rise in median TyG from 8.78 to 9.90, Cluster 2 (rapidly decreasing group, n=239) exhibited a sharp decline from 10.65 to 9.11, Cluster 3 (persistent high group, n=628) maintained stable high TyG levels (10.04 to 10.01), and Cluster 4 (stable low group, n=926) demonstrated a slight TyG decrease from 8.93 to 8.90. Similarly, four heterogeneous TyG trajectories were identified in the private dataset (**Table 2**, **Figures 2D-F**): Cluster 1 (n=157) with TyG increased from 8.52 to 9.42, Cluster 2 (n=145) decreased from 9.49 to 8.53, Cluster 3 (n=369) remained high (9.43 to 9.42), and Cluster 4 (n=595) slightly increased from 8.53 to 8.56. The median TyG values in the private dataset were lower than in MIMIC-IV, while both cohorts exhibited similar metabolic dynamics.

**Table 1:** Baseline characteristics according to TyG trajectory clusters in the MIMIC-IV dataset.

Variable	Overall (n=2038)	Cluster 1 (n=245)	Cluster 2 (n=239)	Cluster 3 (n=628)	Cluster 4 (n=926)	P-value
Hospitalization information						
Hospital mortality	579 (28.41)	95 (38.78)	54 (22.59)	220 (35.03)	210 (22.68)	**<0.001**
Length of stay, days	25.00 (14.00-40.00)	25.00 (18.00-39.00)	27.00 (14.00-43.50)	23.00 (13.00-38.00)	25.00 (14.00-41.00)	0.085
Demographic characters						
Age, years	64.00 (55.00-73.00)	65.00 (55.00-73.00)	61.00 (51.50-68.00)	62.00 (52.00-70.00)	67.00 (58.00-76.00)	**<0.001**
Gender (male)	1283 (62.95)	156 (63.67)	164 (68.62)	401 (63.85)	562 (60.69)	0.133
Laboratory tests						
Triglycerides, mg/dL	209.91 (142.00-306.69)	172.00 (115.00-243.65)	313.37 (203.50-591.46)	269.06 (203.00-386.25)	167.45 (111.03-236.90)	**<0.001**
FBG, mg/dL	133.00 (104.00-179.00)	125.00 (99.00-159.00)	165.00 (120.50-243.00)	155.00 (118.75-218.25)	117.00 (98.00-151.00)	**<0.001**
eGFR, mL/min/1.73m²	47.16 (27.21-71.06)	50.89 (28.32-78.79)	42.04 (23.19-65.60)	46.10 (25.52-71.35)	47.75 (28.35-71.32)	**0.033**
RBC, m/uL	3.58 (3.00-4.16)	3.62 (2.92-4.11)	3.64 (2.95-4.27)	3.66 (3.04-4.24)	3.48 (2.96-4.09)	**0.020**
WBC, K/uL	9.50 (6.80-14.10)	9.20 (6.00-13.20)	9.70 (7.30-13.70)	10.10 (6.97-15.10)	9.30 (6.50-13.88)	**0.042**
Hemoglobin, g/dL	10.50 (8.90-12.20)	10.60 (8.70-12.20)	10.70 (8.85-12.60)	10.65 (8.97-12.40)	10.30 (8.80-12.10)	0.081
RDW, %	15.00 (13.80-16.70)	15.00 (14.10-16.90)	15.00 (13.60-16.30)	14.80 (13.70-16.60)	15.10 (13.80-16.80)	0.054
Hematocrit, %	32.60 (27.62-37.50)	33.10 (27.50-37.10)	32.90 (27.50-38.10)	33.00 (28.08-37.90)	32.00 (27.40-36.90)	0.068
Platelet, K/uL	193.50 (132.00-264.00)	187.00 (128.00-257.00)	199.00 (130.00-262.00)	195.00 (138.00-268.00)	192.50 (131.00-261.75)	0.449
Creatinine, mg/dL	1.40 (1.00-2.30)	1.30 (1.00-2.10)	1.60 (1.20-2.80)	1.50 (1.10-2.50)	1.40 (1.00-2.20)	**<0.001**
Calcium, mg/dL	8.50 (7.90-9.00)	8.50 (8.00-9.00)	8.30 (7.70-8.95)	8.40 (7.80-8.90)	8.50 (8.00-9.10)	**<0.001**
Chloride, mEq/L	102.00 (98.00-106.00)	102.00 (99.00-106.00)	101.00 (97.00-105.00)	102.00 (97.00-106.00)	103.00 (98.00-106.00)	**0.030**
Sodium, mEq/L	138.00 (135.00-141.00)	138.00 (135.00-141.00)	137.00 (132.50-140.00)	137.97 (135.00-140.00)	138.00 (135.00-141.00)	**<0.001**
Potassium, mEq/L	4.20 (3.80-4.70)	4.20 (3.80-4.60)	4.30 (3.80-4.80)	4.30 (3.80-4.80)	4.20 (3.70-4.60)	**0.002**
ALT, IU/L	35.00 (18.00-121.99)	35.00 (18.00-107.00)	34.00 (17.00-109.78)	34.00 (19.00-117.77)	37.00 (16.00-126.00)	0.898
ALP, IU/L	101.85 (68.00-161.57)	98.00 (67.00-153.45)	102.29 (69.00-156.57)	92.50 (66.00-150.03)	110.56 (70.00-170.92)	**0.008**
AST, IU/L	50.00 (24.00-86.77)	45.00 (23.00-84.00)	54.00 (26.00-91.92)	50.00 (26.00-94.12)	51.00 (23.00-82.37)	0.097
BIL, mg/dL	0.70 (0.40-1.34)	0.70 (0.40-1.30)	0.60 (0.40-1.35)	0.70 (0.40-1.30)	0.80 (0.50-1.40)	**0.004**
First TyG	9.36 (8.82-10.02)	8.78 (8.43-9.14)	10.65 (10.02-11.38)	10.04 (9.74-10.48)	8.93 (8.58-9.25)	**<0.001**
Last TyG	9.37 (8.84-9.94)	9.90 (9.60-10.40)	9.11 (8.66-9.71)	10.01 (9.70-10.37)	8.90 (8.52-9.18)	**<0.001**
WATyG	9.45 (8.93-10.04)	9.48 (9.13-10.01)	9.98 (9.41-10.50)	10.06 (9.75-10.45)	8.94 (8.58-9.25)	**<0.001**
Comorbidities						
Diabetic kidney disease	398 (19.53)	48 (19.59)	56 (23.43)	153 (24.36)	141 (15.23)	**<0.001**
CRMS	1261 (61.87)	142 (57.96)	161 (67.36)	437 (69.59)	521 (56.26)	**<0.001**
Hyperlipidemia	1047 (51.37)	130 (53.06)	146 (61.09)	337 (53.66)	434 (46.87)	**<0.001**
Heart failure	701 (34.40)	82 (33.47)	72 (30.13)	208 (33.12)	339 (36.61)	0.213
Ischaemic heart disease	741 (36.36)	87 (35.51)	79 (33.05)	224 (35.67)	351 (37.90)	0.514
Arrhythmia	976 (47.89)	120 (48.98)	97 (40.59)	287 (45.70)	472 (50.97)	**0.019**
Stroke	382 (18.74)	31 (12.65)	48 (20.08)	102 (16.24)	201 (21.71)	**0.003**
PVD	682 (33.46)	79 (32.24)	93 (38.91)	185 (29.46)	325 (35.10)	**0.030**
Diabetes	927 (45.49)	97 (39.59)	149 (62.34)	360 (57.32)	321 (34.67)	**<0.001**
Respiratory failure	1046 (51.32)	146 (59.59)	125 (52.30)	348 (55.41)	427 (46.11)	**<0.001**
Cancer	422 (20.71)	65 (26.53)	35 (14.64)	115 (18.31)	207 (22.35)	**0.003**
COPD	1056 (51.82)	143 (58.37)	118 (49.37)	372 (59.24)	423 (45.68)	**<0.001**
Anemia	1308 (64.18)	164 (66.94)	150 (62.76)	395 (62.90)	599 (64.69)	0.666
Electrolyte Disturbance	1528 (74.98)	204 (83.27)	192 (80.33)	507 (80.73)	625 (67.49)	**<0.001**
Drugs						
**Anti-hypertensive drugs**	1870 (91.76)	224 (91.43)	222 (92.89)	585 (93.15)	839 (90.60)	0.298
ACEI/ARB	495 (24.29)	49 (20.00)	56 (23.43)	134 (21.34)	256 (27.65)	**0.011**
Loop Diuretics	1489 (73.06)	182 (74.29)	169 (70.71)	499 (79.46)	639 (69.01)	**<0.001**
Beta blockers	1462 (71.74)	171 (69.80)	173 (72.38)	445 (70.86)	673 (72.68)	0.766
Calcium channel blockers	150 (7.36)	16 (6.53)	26 (10.88)	53 (8.44)	55 (5.94)	**0.038**
Other anti-hypertensive drugs	749 (36.75)	88 (35.92)	92 (38.49)	223 (35.51)	346 (37.37)	0.813
**Anti-platelet drugs**	980 (48.09)	115 (46.94)	124 (51.88)	277 (44.11)	464 (50.11)	0.072
Aspirin	948 (46.52)	115 (46.94)	119 (49.79)	268 (42.68)	446 (48.16)	0.123
Other anti-platelet drugs	238 (11.68)	26 (10.61)	34 (14.23)	59 (9.39)	119 (12.85)	0.103
**Hypoglycemic drugs**	1636 (80.27)	205 (83.67)	215 (89.96)	568 (90.45)	648 (69.98)	**<0.001**
Insulin	1636 (80.27)	205 (83.67)	215 (89.96)	568 (90.45)	648 (69.98)	**<0.001**
Oral hypoglycemic drugs	97 (4.76)	5 (2.04)	25 (10.46)	31 (4.94)	36 (3.89)	**<0.001**
**Lipid-modifying drugs**	1117 (54.81)	121 (49.39)	153 (64.02)	359 (57.17)	484 (52.27)	**0.002**
Statins	1009 (49.51)	112 (45.71)	130 (54.39)	309 (49.20)	458 (49.46)	0.294
Other lipid-modifying drugs	242 (11.87)	29 (11.84)	49 (20.50)	102 (16.24)	62 (6.70)	**<0.001**

Bold values indicate statistical significance (P < 0.05).

**Table 2:** Baseline characteristics according to TyG trajectory clusters in the private dataset.

Variable	Overall (n=1266)	Cluster 1 (n=157)	Cluster 2 (n=145)	Cluster 3 (n=369)	Cluster 4 (n=595)	P-value
Hospitalization information						
Hospital mortality	89 (7.03)	29 (18.47)	8 (5.52)	28 (7.59)	24 (4.03)	**<0.001**
Length of stay, days	12.00 (9.00-16.00)	11.00 (8.00-14.00)	12.00 (8.00-15.00)	12.00 (9.00-15.00)	13.00 (10.00-17.00)	**<0.001**
Demographic characters						
Age, years	74.00 (62.00-81.00)	74.00 (65.00-80.00)	73.00 (60.00-80.00)	69.00 (58.00-79.00)	77.00 (66.00-82.00)	**<0.001**
Gender (male)	806 (63.67)	100 (63.69)	76 (52.41)	225 (60.98)	405 (68.07)	**0.003**
Laboratory tests						
Triglycerides, mg/dL	124.88 (89.68-179.58)	95.66 (69.97-132.85)	176.25 (119.57-243.57)	185.11 (139.05-250.65)	103.63 (76.76-131.31)	**<0.001**
FBG, mg/dL	110.16 (90.72-145.84)	107.82 (86.94-139.50)	138.78 (104.94-237.60)	132.48 (105.84-178.38)	97.92 (85.68-117.45)	**<0.001**
eGFR, mL/min/1.73m²	42.76 (23.17-56.45)	43.41 (21.29-60.00)	40.46 (19.47-50.17)	41.05 (23.04-57.84)	43.54 (24.44-56.44)	0.335
RBC, m/uL	3.72 (3.16-4.26)	3.73 (3.12-4.22)	3.85 (3.40-4.31)	3.75 (3.28-4.36)	3.69 (3.08-4.21)	**0.016**
WBC, K/uL	7.19 (5.70-9.42)	7.47 (6.14-10.29)	7.56 (6.04-10.06)	7.55 (6.12-10.05)	6.58 (5.34-8.65)	**<0.001**
Hemoglobin, g/dL	11.19 (9.50-12.90)	10.80 (9.10-12.60)	11.40 (10.20-12.80)	11.30 (9.70-13.10)	11.10 (9.30-12.90)	0.107
RDW, %	13.60 (12.90-14.50)	13.80 (13.10-14.70)	13.50 (13.00-14.60)	13.40 (12.80-14.40)	13.60 (13.00-14.50)	**0.029**
Hematocrit, %	33.63 (28.40-38.20)	32.90 (27.60-37.20)	34.10 (30.90-38.20)	33.70 (29.20-38.50)	33.60 (27.95-38.25)	0.173
Platelet, K/uL	196.50 (156.00-246.72)	196.00 (156.00-248.41)	203.00 (159.00-252.00)	199.00 (161.00-257.37)	193.00 (153.00-241.00)	0.113
Creatinine, mg/dL	1.55 (1.23-2.53)	1.52 (1.22-2.67)	1.69 (1.22-2.69)	1.61 (1.23-2.58)	1.53 (1.24-2.34)	0.589
Calcium, mg/dL	2.10 (1.98-2.22)	2.05 (1.94-2.18)	2.12 (1.96-2.23)	2.11 (1.98-2.24)	2.10 (1.98-2.22)	**0.037**
Chloride, mEq/L	102.70 (99.50-105.50)	102.60 (98.60-105.80)	100.90 (97.60-103.50)	102.70 (99.80-105.80)	103.20 (99.92-105.69)	**<0.001**
Sodium, mEq/L	140.39 (137.50-142.50)	140.30 (137.50-142.90)	138.80 (136.00-142.00)	140.50 (137.80-142.70)	140.40 (137.80-142.35)	**0.010**
Potassium, mEq/L	4.24 (3.85-4.68)	4.05 (3.75-4.61)	4.36 (3.95-4.79)	4.21 (3.83-4.58)	4.25 (3.87-4.72)	**0.002**
ALT, IU/L	13.00 (9.00-21.00)	15.00 (8.00-24.78)	16.00 (10.00-25.00)	14.00 (9.00-24.00)	12.00 (8.00-18.50)	**<0.001**
ALP, IU/L	65.00 (54.00-81.00)	68.00 (54.00-85.00)	69.00 (55.00-87.00)	66.00 (53.00-81.00)	64.00 (53.00-77.00)	0.065
AST, IU/L	19.00 (15.00-28.00)	22.00 (17.00-35.00)	20.00 (16.00-30.00)	19.00 (15.00-29.00)	18.00 (15.00-25.00)	**<0.001**
BIL, mg/dL	8.71 (6.25-12.59)	8.91 (6.27-13.23)	8.75 (6.07-11.87)	8.52 (6.15-12.39)	8.88 (6.29-12.81)	0.695
First TyG	8.86 (8.43-9.36)	8.52 (8.08-8.84)	9.49 (9.11-9.92)	9.43 (9.17-9.77)	8.53 (8.26-8.80)	**<0.001**
Last TyG	8.88 (8.45-9.33)	9.42 (8.97-9.80)	8.53 (8.18-8.97)	9.42 (9.15-9.78)	8.56 (8.24-8.81)	**<0.001**
WATyG	8.88 (8.49-9.29)	8.94 (8.54-9.27)	9.00 (8.71-9.41)	9.43 (9.22-9.76)	8.57 (8.27-8.80)	**<0.001**
Comorbidities						
Diabetic kidney disease	174 (13.74)	23 (14.65)	26 (17.93)	71 (19.24)	54 (9.08)	**<0.001**
CRMS	690 (54.50)	86 (54.78)	97 (66.90)	263 (71.27)	244 (41.01)	**<0.001**
Hyperlipidemia	133 (10.51)	11 (7.01)	27 (18.62)	56 (15.18)	39 (6.55)	**<0.001**
Heart failure	438 (34.60)	68 (43.31)	47 (32.41)	114 (30.89)	209 (35.13)	**0.048**
Ischaemic heart disease	595 (47.00)	79 (50.32)	59 (40.69)	167 (45.26)	290 (48.74)	0.242
Arrhythmia	253 (19.98)	38 (24.20)	26 (17.93)	60 (16.26)	129 (21.68)	0.094
Stroke	422 (33.33)	56 (35.67)	56 (38.62)	108 (29.27)	202 (33.95)	0.168
PVD	228 (18.01)	21 (13.38)	36 (24.83)	56 (15.18)	115 (19.33)	**0.023**
Diabetes	631 (49.84)	82 (52.23)	89 (61.38)	243 (65.85)	217 (36.47)	**<0.001**
Respiratory failure	95 (7.50)	23 (14.65)	9 (6.21)	30 (8.13)	33 (5.55)	**0.002**
Cancer	97 (7.66)	17 (10.83)	7 (4.83)	18 (4.88)	55 (9.24)	**0.018**
COPD	364 (28.75)	69 (43.95)	36 (24.83)	98 (26.56)	161 (27.06)	**<0.001**
Anemia	451 (35.62)	60 (38.22)	50 (34.48)	124 (33.60)	217 (36.47)	0.709
Electrolyte Disturbance	231 (18.25)	37 (23.57)	29 (20.00)	75 (20.33)	90 (15.13)	**0.041**
Drugs						
**Anti-hypertensive drugs**	964 (76.15)	117 (74.52)	96 (66.21)	290 (78.59)	461 (77.48)	**0.019**
ACEI/ARB	532 (42.02)	48 (30.57)	53 (36.55)	170 (46.07)	261 (43.87)	**0.004**
Loop Diuretics	334 (26.38)	56 (35.67)	30 (20.69)	101 (27.37)	147 (24.71)	**0.015**
Beta blockers	516 (40.76)	53 (33.76)	55 (37.93)	169 (45.80)	239 (40.17)	0.054
Calcium channel blockers	85 (6.71)	8 (5.10)	9 (6.21)	32 (8.67)	36 (6.05)	0.335
Other anti-hypertensive drugs	761 (60.11)	97 (61.78)	65 (44.83)	223 (60.43)	376 (63.19)	**<0.001**
**Anti-platelet drugs**	548 (43.29)	63 (40.13)	53 (36.55)	176 (47.70)	256 (43.03)	0.100
Aspirin	444 (35.07)	55 (35.03)	47 (32.41)	140 (37.94)	202 (33.95)	0.549
Other anti-platelet drugs	310 (24.49)	35 (22.29)	31 (21.38)	104 (28.18)	140 (23.53)	0.242
**Hypoglycemic drugs**	591 (46.68)	87 (55.41)	75 (51.72)	224 (60.70)	205 (34.45)	**<0.001**
Insulin	500 (39.49)	82 (52.23)	60 (41.38)	190 (51.49)	168 (28.24)	**<0.001**
Oral hypoglycemic drugs	302 (23.85)	39 (24.84)	36 (24.83)	120 (32.52)	107 (17.98)	**<0.001**
**Lipid-modifying drugs**	551 (43.52)	70 (44.59)	62 (42.76)	182 (49.32)	237 (39.83)	**0.038**
Statins	545 (43.05)	69 (43.95)	60 (41.38)	180 (48.78)	236 (39.66)	**0.047**
Other lipid-modifying drugs	16 (1.26)	2 (1.27)	3 (2.07)	10 (2.71)	1 (0.17)	**0.005**

Bold values indicate statistical significance (P < 0.05).

In MIMIC-IV, 1,283 patients (62.95%) were male, with a median age of 64 years; in the private dataset, 806 patients (63.67%) were male, with a median age of 74 years. In-hospital mortality rates were 28.41% (n=579, MIMIC-IV) and7.03% (n=89, private dataset), respectively. Clusters 1 (rapidly increasing TyG) and 3 (persistent high TyG) exhibited significantly higher mortality rates than Clusters 2 (rapidly decreasing TyG) and 4 (stable low TyG) in both datasets (MIMIC-IV: 38.78%/35.03% vs. 22.59%/22.68%; private dataset: 18.47%/7.59% vs. 5.52%/4.03%; all P<0.001). In MIMIC-IV, the overall median TyG increased slightly from 9.36 (first measurement) to 9.37 (last measurement), with a median WATyG of 9.45; in the private dataset, it increased from 8.86 to 8.88, with a median WATyG of 8.88.

In both datasets, Clusters 1, 2 and 3 consistently had higher comorbidity prevalence than Cluter 4 (stable low TyG). The comorbidities showing this consistent pattern across both datasets included DKD, CRMS, hyperlipidemia, diabetes, and COPD, all of which achieved statistical significance (all P< 0.001). As representative examples, DKD and CRMS exhibited aligned cluster-specific distributions across the two datasets. In MIMIC-IV, DKD had an overall prevalence of 19.53% (n=398), with Cluster 3 (24.36%) and Cluster 2 (23.43%) recording the highest rates, while Cluster 4 had the lowest (15.23%); for CRMS (overall prevalence 61.87%, n=1261), Cluster 3 (69.59%) and Cluster 2 (67.36%) also ranked highest, significantly exceeding Cluster 4 (56.26%). The private dataset reflected this pattern consistently, with DKD (overall prevalence 13.74%, n=174) being the most prevalent in Cluster 3 (19.24%) and Cluster 2 (17.93%), in contrast to the lowest rate in Cluster 4 (9.08%); CRMS (overall prevalence 54.50%, n=690) followed the same hierarchy, as Cluster 3 (71.27%) and Cluster 2 (66.90%) had notably higher prevalence than Cluster 4 (41.01%).

For laboratory indicators, TG and FBG are key metabolic markers linked to TyG indices, and they were consistently elevated in Clusters 2 and 3 relative to Cluster 4 in both datasets (all P< 0.001). This finding reflects a shared association between higher TyG trajectories and perturbed glucose-lipid metabolism across the two datasets. Beyond these metabolic parameters, in both datasets, WBC were higher in Cluster 3 than in Cluster 4, while sodium, potassium and calcium levels were lower in Cluster 2 compared to Cluster 4 (all P<0.05), and potassium levels also followed a consistent trend as they were higher in Clusters 2 and 3 than in Cluster 4 (both P<0.05). Cross-dataset consistencies indicate that TyG trajectory clusters correlate with distinct biochemical profiles, and metabolic and electrolyte abnormalities concentrate in groups with higher or rapidly changing TyG levels.

Medications including hypoglycemic drugs (insulin), lipid-modifying drugs, and loop diuretics exhibited significant between-cluster differences with consistent usage trends across both datasets (all P< 0.05). In contrast, other drug classes such as beta blockers and aspirin showed no significant between-cluster differences in either dataset (all P > 0.05). Hypoglycemic drugs (including insulin) were most frequently used in Clusters 2 and 3 (higher TyG levels) compared to Cluster 4. In MIMIC-IV, hypoglycemic drug usage reached 89.96% and 90.45% in Clusters 2 and 3 versus 69.98% in Cluster 4 (P< 0.001), while in private dataset, hypoglycemic drug usage was 51.72% and 60.70% in Clusters 2 and 3 versus 28.24% in Cluster 4 (P< 0.001). Lipid-modifying drugs followed a similar pattern, with usage 64.02% and 57.17% in Clusters 2 and 3 of MIMIC-IV (vs. 52.27% in Cluster 4, P=0.002) and 49.32%in Cluster 3 of the private dataset (vs. 39.83% in Cluster 4, P=0.038). Loop diuretics were also more commonly used in Clusters 1 and 3 relative to Cluster 4, with significant differences in both datasets (MIMIC-IV: 74.29% and 79.46% vs. 69.01%, P< 0.001; private: 35.67% and 27.37% vs. 24.71%, P=0.015). These findings indicate that the observed medication patterns are closely tied to the metabolic and renal characteristics reflected by TyG trajectories.

### 3.2 TyG dynamic trajectories, WATyG and mortality

**Table 3** presents logistic regression results investigating the association between TyG trajectories, WATyG, and mortality in patients with hypertension and KF. All analyses adopted Cluster 1 as the reference group.

**Table 3:** Logistic regression analysis of TyG trajectories, time-weighted average TyG (WATyG) and mortality in patients with hypertension and kidney failure^1^.

		Model 1		Model 2		Model 3		Model 4	
Datasets	Variables	P-value	OR (95% CI)	P-value	OR (95% CI)	P-value	OR (95% CI)	P-value	OR (95% CI)
The MIMIC-IV dataset	TyG trajectories								
	**Cluster 1**	Reference		Reference		Reference		Reference	
	**Cluster 2**	**<0.001**	0.461 (0.310-0.686)	**<0.001**	0.486 (0.326-0.726)	**0.004**	0.525 (0.339-0.814)	**0.007**	0.546 (0.351-0.849)
	**Cluster 3**	0.301	0.851 (0.628-1.155)	0.441	0.886 (0.652-1.205)	0.689	0.933 (0.667-1.307)	0.702	0.936 (0.666-1.315)
	**Cluster 4**	**<0.001**	0.463 (0.343-0.625)	**<0.001**	0.433 (0.320-0.587)	**<0.001**	0.486 (0.349-0.677)	**<0.001**	0.492 (0.352-0.687)
	**WATyG**	**<0.001**	1.340 (1.188-1.511)	**<0.001**	1.469 (1.294-1.668)	**<0.001**	1.505 (1.297-1.746)	**<0.001**	1.505 (1.293-1.752)
	Quartile of WATyG								
	**Q1**	Reference		Reference		Reference		Reference	
	**Q2**	**0.003**	1.556 (1.167-2.073)	**0.001**	1.652 (1.236-2.208)	**0.006**	1.542 (1.129-2.106)	**0.012**	1.495 (1.092-2.046)
	**Q3**	**0.002**	1.575 (1.183-2.097)	**<0.001**	1.777 (1.327-2.380)	**0.004**	1.593 (1.161-2.187)	**0.006**	1.562 (1.134-2.153)
	**Q4**	**<0.001**	2.173 (1.643-2.875)	**<0.001**	2.661 (1.985-3.567)	**<0.001**	2.579 (1.853-3.588)	**<0.001**	2.554 (1.825-3.573)
The private dataset	TyG trajectories								
	**Cluster 1**	Reference		Reference		Reference		Reference	
	**Cluster 2**	**0.001**	0.258 (0.114-0.585)	**0.002**	0.274 (0.120-0.627)	**0.015**	0.286 (0.105-0.780)	**0.012**	0.255 (0.088-0.737)
	**Cluster 3**	**<0.001**	0.362 (0.208-0.633)	**0.001**	0.398 (0.227-0.700)	0.084	0.539 (0.267-1.088)	0.081	0.519 (0.249-1.084)
	**Cluster 4**	**<0.001**	0.186 (0.105-0.329)	**<0.001**	0.175 (0.098-0.312)	**<0.001**	0.242 (0.120-0.487)	**0.001**	0.308 (0.150-0.635)
	**WATyG**	**0.002**	1.630 (1.193-2.228)	**<0.001**	1.887 (1.363-2.614)	**0.013**	1.730 (1.122-2.667)	0.249	1.313 (0.827-2.084)
	Quartile of WATyG								
	**Q1**	Reference		Reference		Reference		Reference	
	**Q2**	0.106	1.749 (0.888-3.446)	0.078	1.845 (0.933-3.648)	0.052	2.227 (0.994-4.991)	0.211	1.636 (0.756-3.539)
	**Q3**	0.339	1.413 (0.696-2.870)	0.156	1.685 (0.820-3.461)	0.249	1.662 (0.701-3.940)	0.501	1.329 (0.581-3.041)
	**Q4**	**0.007**	2.455 (1.284-4.697)	**0.001**	3.089 (1.583-6.028)	**0.011**	2.917 (1.278-6.660)	**0.023**	2.483 (1.134-5.437)

^1^Model 1 was unadjusted. Model 2 was adjusted for age and gender. Model 3 was adjusted for all variables in Model 2, plus additional comorbidities (DKD, CRMS, hyperlipidemia, heart failure, ischaemic heart disease, arrhythmia, stroke, peripheral vascular disease, diabetes, respiratory failure, cancer, anemia, electrolyte disturbance and COPD) and laboratory indicators (TG, FBG, eGFR, RBC, WBC, haemoglobin, RDW, haematocrit, platelet, creatinine, Ca, Cl, Na, K, ALT, ALP, AST and BIL). Model 4 was adjusted for all variables in Model 3, plus additional anti-hypertensive drugs, anti-platelet drugs, hypoglycemic drugs and lipid-modifying drugs. Bold values indicate statistical significance (P < 0.05).

In the MIMIC-IV dataset, the fully adjusted model 4 showed that Cluster 1 had a significantly higher mortality risk than Cluster 2 and Cluster 4. The OR for Cluster 2 was 0.546 (95%CI 0.351-0.849, P=0.007). The OR for Cluster 4 was 0.492 (95%CI 0.352-0.687, P< 0.001). No significant difference in mortality risk was observed between Cluster 1 and Cluster 3 (P=0.702). As a continuous variable, WATyG was independently associated with increased mortality risk, with an OR of 1.505 (95%CI 1.293-1.752, P< 0.001). When WATyG was categorized into quartiles, higher quartiles exhibited progressively elevated mortality risks relative to the lowest quartile (Q1). In MIMIC-IV, the OR for Q2 was 1.495 (95% CI 1.092-2.046, P=0.012), for Q3 it was 1.562 (95% CI 1.134-2.153, P=0.006) and for Q4 it was 2.554 (95% CI 1.825-3.573, P< 0.001). In the private dataset, the fully adjusted model also indicated significantly lower mortality risks in Clusters 2 and 4 compared to Cluster 1. The OR for Cluster 2 was 0.255 (95% CI 0.088-0.737, P=0.012), and for Cluster 4 it was 0.308 (95% CI 0.150-0.635, P=0.001). The association between WATyG (as a continuous variable) and mortality was not statistically significant in private dataset (P=0.249). However, the highest quartile (Q4) of WATyG still showed a significant mortality risk increase relative to Q1 (OR 2.483, 95% CI 1.134-5.437, P=0.023).

These findings from the fully adjusted model that accounts for medication use consistently confirm that TyG trajectory clusters and WATyG levels are closely linked to mortality risk in patients with hypertension and KF across both datasets. Cluster 1 consistently presents a higher mortality risk than Clusters 2 and 4. Higher WATyG quartiles, especially Q4, are associated with elevated mortality risk even after accounting for the influence of drugs.

**Figure 3:**
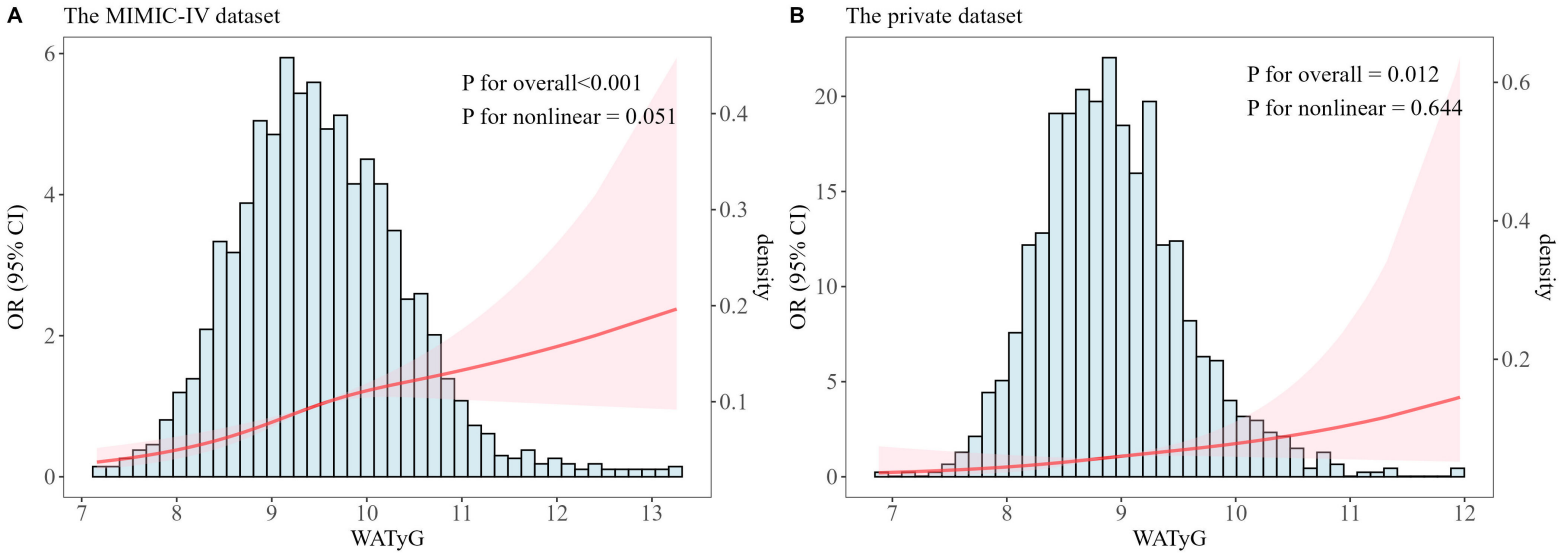
Restricted cubic spline (RCS) analysis of WATyG-mortality association in patients with hypertension and kidney failure (KF) from the MIMIC-IV **(A)** and the private datasets **(B)**. RCS models were adjusted by age, gender, comorbidities (DKD, CRMS, hyperlipidemia, heart failure, ischaemic heart disease, arrhythmia, stroke, peripheral vascular disease, diabetes, respiratory failure, cancer, anemia, electrolyte disturbance and COPD), laboratory indicators (TG, FBG, eGFR, RBC, WBC, haemoglobin, RDW, haematocrit, platelet, creatinine, Ca, Cl, Na, K, ALT, ALP, AST and BIL), and anti-hypertensive drugs, anti-platelet drugs, hypoglycemic drugs and lipid-modifying drugs.

Multivariable-adjusted RCS analysis showed significant linear associations between WATyG and mortality in both datasets (**Figure 3**; P for overall< 0.05, P for nonlinear > 0.05). Linear associations were also observed across four TyG trajectory clusters in MIMIC-IV (**Supplementary Figure S1**; all P for nonlinear > 0.05) and the private dataset (**Supplementary Figure S2**; all P for nonlinear > 0.05).

### 3.3 Subgroup analyses

**Table 4** (MIMIC-IV) and **Supplementary Table S1** (private dataset) present subgroup analyses of the association between TyG trajectories and mortality risk in patients with hypertension and KF. These analyses were stratified by age, gender, comorbidities and drug use that are clinically relevant to this population. Notable interaction effects emerged for age and respiratory failure. In MIMIC-IV, significant interactions were observed between TyG trajectories and age (P for interaction=0.044) as well as respiratory failure (P for interaction=0.009). The private dataset also showed a significant interaction between TyG trajectories and age (P for interaction=0.024). In contrast, no significant interactions were found for CRMS, DKD or hyperlipidemia in either dataset (all P for interaction > 0.05). This finding indicates that the association between TyG trajectories and mortality remained consistent across most clinically important subgroups.

**Table 4:** Subgroup analysis of the association between TyG trajectories and mortality risks in patients with hypertension and kidney failure in the MIMIC-IV dataset.

Variable	Cluster 1	Cluster 2	Cluster 3	Cluster 4	P for Trend	P for interaction
**Age**						**0.044**
<65	Reference	0.455 (0.241-0.861)	0.891 (0.548-1.448)	0.621 (0.378-1.021)	0.311	
≥65	Reference	0.618 (0.329-1.164)	1.009 (0.618-1.648)	0.410 (0.258-0.651)	**<0.001**	
**Gender**						0.868
Female	Reference	0.386 (0.173-0.859)	1.034 (0.580-1.844)	0.461 (0.263-0.808)	**0.027**	
Male	Reference	0.592 (0.345-1.016)	0.868 (0.567-1.329)	0.548 (0.359-0.835)	**0.018**	
**Cardio-renal-metabolic syndrome**						0.331
No	Reference	0.578 (0.265-1.262)	0.967 (0.549-1.702)	0.600 (0.354-1.018)	0.101	
Yes	Reference	0.476 (0.275-0.824)	0.826 (0.536-1.272)	0.464 (0.299-0.720)	**0.006**	
**Diabetic kidney disease**						0.464
No	Reference	0.574 (0.351-0.940)	0.837 (0.573-1.224)	0.491 (0.341-0.709)	**<0.001**	
Yes	Reference	0.330 (0.117-0.930)	1.118 (0.509-2.455)	0.564 (0.240-1.323)	0.723	
**Hyperlipidemia**						0.053
No	Reference	0.560 (0.286-1.098)	0.945 (0.571-1.565)	0.637 (0.394-1.030)	0.146	
Yes	Reference	0.425 (0.232-0.779)	0.864 (0.540-1.383)	0.377 (0.233-0.611)	**0.001**	
**Ischaemic heart disease**						0.984
No	Reference	0.384 (0.216-0.682)	0.723 (0.468-1.117)	0.451 (0.294-0.692)	**0.006**	
Yes	Reference	0.694 (0.337-1.428)	1.248 (0.710-2.195)	0.544 (0.312-0.949)	**0.034**	
**Peripheral vascular disease**						0.391
No	Reference	0.508 (0.290-0.889)	0.879 (0.584-1.322)	0.468 (0.310-0.704)	**0.002**	
Yes	Reference	0.505 (0.238-1.074)	0.938 (0.501-1.757)	0.586 (0.323-1.066)	0.212	
**Respiratory failure**						**0.009**
No	Reference	0.550 (0.263-1.151)	0.851 (0.471-1.540)	0.317 (0.176-0.569)	**<0.001**	
Yes	Reference	0.423 (0.241-0.741)	0.755 (0.492-1.158)	0.577 (0.380-0.876)	0.091	
**Heart failure**						0.743
No	Reference	0.573 (0.332-0.988)	0.902 (0.590-1.379)	0.546 (0.358-0.833)	**0.019**	
Yes	Reference	0.400 (0.185-0.865)	0.820 (0.461-1.459)	0.407 (0.233-0.712)	**0.007**	
**Arrhythmia**						0.689
No	Reference	0.442 (0.231-0.845)	0.679 (0.411-1.122)	0.458 (0.278-0.755)	**0.015**	
Yes	Reference	0.529 (0.286-0.978)	1.043 (0.652-1.667)	0.528 (0.334-0.834)	**0.017**	
**Diabetes**						0.997
No	Reference	0.468 (0.242-0.906)	0.920 (0.580-1.459)	0.501 (0.326-0.768)	**0.005**	
Yes	Reference	0.571 (0.306-1.066)	0.947 (0.564-1.590)	0.540 (0.313-0.932)	0.103	
**Stroke**						0.380
No	Reference	0.478 (0.292-0.782)	0.860 (0.597-1.239)	0.522 (0.364-0.749)	**0.005**	
Yes	Reference	0.731 (0.239-2.232)	1.165 (0.440-3.086)	0.438 (0.172-1.114)	**0.037**	
**Electrolyte Disturbance**						**0.038**
No	Reference	0.192 (0.046-0.796)	0.617 (0.223-1.705)	0.163 (0.060-0.443)	**<0.001**	
Yes	Reference	0.545 (0.342-0.869)	0.884 (0.616-1.268)	0.588 (0.412-0.839)	**0.024**	
**Cancer**						0.667
No	Reference	0.447 (0.272-0.735)	0.725 (0.491-1.070)	0.457 (0.311-0.670)	**0.001**	
Yes	Reference	1.060 (0.362-3.109)	1.308 (0.591-2.892)	0.734 (0.350-1.541)	0.325	
**Chronic obstructive pulmonary disease**						0.672
No	Reference	0.332 (0.157-0.700)	0.456 (0.248-0.838)	0.409 (0.232-0.719)	**0.027**	
Yes	Reference	0.530 (0.302-0.929)	0.996 (0.653-1.520)	0.494 (0.323-0.754)	**0.007**	
**Anemia**						0.234
No	Reference	0.290 (0.124-0.674)	0.835 (0.451-1.546)	0.338 (0.180-0.632)	**0.011**	
Yes	Reference	0.630 (0.372-1.066)	0.853 (0.564-1.290)	0.602 (0.404-0.897)	**0.03**	
**Anti-hypertensive drugs**						0.158
No	Reference	0.578 (0.025-13.170)	1.923 (0.207-17.883)	0.193 (0.027-1.395)	0.073	
Yes	Reference	0.529 (0.335-0.835)	0.871 (0.613-1.237)	0.554 (0.392-0.782)	**0.007**	
**Anti-platelet drugs**						0.187
No	Reference	0.468 (0.249-0.879)	0.966 (0.604-1.544)	0.548 (0.345-0.871)	0.053	
Yes	Reference	0.530 (0.280-1.004)	0.799 (0.482-1.325)	0.448 (0.272-0.736)	**0.005**	
**Hypoglycemic drugs**						0.931
No	Reference	0.204 (0.044-0.952)	0.993 (0.325-3.031)	0.366 (0.141-0.949)	0.085	
Yes	Reference	0.533 (0.335-0.848)	0.885 (0.617-1.269)	0.523 (0.363-0.752)	**0.005**	
**Lipid-modifying drugs**						**0.002**
No	Reference	0.618 (0.308-1.240)	1.382 (0.839-2.279)	0.831 (0.513-1.345)	0.736	
Yes	Reference	0.429 (0.237-0.774)	0.643 (0.399-1.037)	0.329 (0.204-0.531)	**<0.001**	

Bold values indicate statistical significance (P < 0.05).

In terms of subgroup-specific associations the MIMIC-IV dataset revealed significant links between TyG trajectories and mortality in patients aged ≥65 years (P for trend< 0.001), both males and females (both P for trend< 0.05). Significant associations were also observed in patients with CRMS (P for trend=0.006) where Clusters 2, Cluster 3 and 4 had reduced mortality risk relative to Cluster 1 (OR 0.476, 0.826 and 0.464 respectively). The private dataset showed significant associations between TyG trajectories and mortality in males (P for trend=0.021) and patients without CRMS (P for trend =0.009), without DKD (P for trend=0.023), without hyperlipidemia (P for tend=0.010). In males Cluster 4 had lower mortality risk than Cluster 1 (OR 0.286). In patients without DKD, Cluster 2 (OR 0.179), Cluster 3 (OR 0.534) and Cluster 4 (OR 0.325) exhibited reduced mortality risk compared to Cluster 1.

These results confirm the robustness of the association between TyG trajectories and mortality risk in patients with hypertension and KF. The consistency across subgroups defined by CRMS and DKD highlights the reliability of this association. While subtle interaction effects for age and respiratory failure suggest the strength of the association may vary by these factors the overall directional trend remains stable. Specifically, Clusters 2 and 4 consistently show lower mortality risk than Cluster 1, which supports the potential of TyG trajectories as a reliable mortality predictor in this population.

**Table 5** (continuous WATyG) and **Supplementary Tables S2**-**S3** (WATyG quartiles) present subgroup analyses of the association between WATyG and mortality risk in patients with hypertension and KF. Key interaction effects were consistent across continuous and quartile-based WATyG analyses in the MIMIC-IV dataset, with significant interactions observed for age (continuous: P for interaction=0.007; quartiles: P for interaction =0.003), respiratory failure (both P for interaction<0.001), and COPD (both P for interaction =0.017). The private dataset showed distinct significant interactions, including gender for continuous WATyG (P for interaction =0.031) and respiratory failure for WATyG quartiles (P for interaction =0.003). No significant interactions were found for CRMS, DKD or hyperlipidemia in either dataset (all P for interaction >0.05), confirming consistent WATyG-mortality associations across these subgroups.

**Table 5:** Subgroup analysis of the association between WATyG and mortality in patients with hypertension and kidney failure.

Variable	The MIMIC-IV dataset			The private dataset		
	OR (95% CI)	P for Trend	P for interaction	OR (95% CI)	P for Trend	P for interaction
**Age**			**0.007**			0.762
<65	1.277 (1.035-1.576)	**0.023**		1.503 (0.429-5.272)	0.524	
≥65	1.817 (1.449-2.279)	**<0.001**		1.901 (1.161-3.112)	**0.011**	
**Gender**			0.280			0.031
Female	1.514 (1.173-1.954)	**0.001**		0.763 (0.349-1.670)	0.499	
Male	1.314 (1.095-1.577)	**0.003**		2.328 (1.352-4.008)	**0.002**	
**Cardio-renal-metabolic syndrome**			0.164			0.862
No	1.256 (0.974-1.620)	0.079		1.592 (0.860-2.946)	0.139	
Yes	1.420 (1.183-1.705)	**<0.001**		1.625 (0.879-3.004)	0.122	
**Diabetic kidney disease**			0.639			0.147
No	1.420 (1.203-1.678)	**<0.001**		1.437 (0.931-2.219)	0.102	
Yes	1.321 (0.942-1.853)	0.107		Inf (0.000-Inf)	1.000	
**Hyperlipidemia**			0.059			0.153
No	1.229 (0.986-1.531)	0.067		1.680 (1.104-2.556)	**0.015**	
Yes	1.492 (1.218-1.829)	**<0.001**		0.000 (0.000-Inf)	1.000	
**Ischaemic heart diseases**			0.477			0.073
No	1.381 (1.142-1.670)	**<0.001**		1.066 (0.584-1.946)	0.834	
Yes	1.427 (1.123-1.814)	**0.004**		2.503 (1.291-4.853)	**0.007**	
**Peripheral vascular disease**			0.767			0.435
No	1.406 (1.173-1.685)	**<0.001**		1.831 (1.174-2.856)	**0.008**	
Yes	1.323 (1.027-1.703)	**0.03**		3.011 (0.402-22.563)	0.283	
**Respiratory failure**			**<0.001**			0.118
No	1.968 (1.531-2.530)	**<0.001**		1.670 (0.973-2.867)	0.063	
Yes	1.080 (0.895-1.303)	0.421		0.197 (0.047-0.826)	**0.026**	
**Heart failure**			0.976			0.369
No	1.388 (1.153-1.670)	**<0.001**		1.257 (0.692-2.283)	0.453	
Yes	1.374 (1.068-1.768)	**0.014**		2.163 (1.127-4.151)	**0.020**	
**Arrhythmia**			0.311			0.838
No	1.270 (1.020-1.583)	**0.033**		1.499 (0.892-2.521)	0.127	
Yes	1.485 (1.209-1.825)	**<0.001**		1.692 (0.743-3.851)	0.210	
**Diabetes**			0.101			0.722
No	1.617 (1.302-2.008)	**<0.001**		1.599 (0.879-2.910)	0.124	
Yes	1.212 (0.985-1.491)	0.069		1.753 (0.916-3.354)	0.090	
**Stroke**			0.258			0.167
No	1.372 (1.167-1.614)	**<0.001**		1.293 (0.755-2.214)	0.350	
Yes	1.602 (1.085-2.364)	**0.018**		5.000 (2.004-12.476)	**<0.001**	
**Electrolyte Disturbance**			0.059			0.125
No	1.969 (1.263-3.069)	**0.003**		2.091 (1.164-3.758)	**0.014**	
Yes	1.295 (1.107-1.515)	**0.001**		0.857 (0.425-1.730)	0.668	
**Cancer**			**0.011**			0.322
No	1.240 (1.052-1.462)	**0.01**		1.664 (1.089-2.544)	**0.019**	
Yes	2.046 (1.407-2.975)	**<0.001**		0.000 (0.000-Inf)	1.000	
**Chronic obstructive pulmonary disease**			**0.017**			0.675
No	1.092 (0.862-1.383)	0.467		1.440 (0.705-2.942)	0.317	
Yes	1.516 (1.245-1.846)	**<0.001**		1.418 (0.801-2.511)	0.231	
**Anemia**			0.255			0.283
No	1.538 (1.173-2.017)	**0.002**		1.516 (0.932-2.468)	0.094	
Yes	1.302 (1.089-1.557)	**0.004**		4.135 (1.313-13.027)	**0.015**	
**Anti-hypertensive drugs**			**0.004**			0.065
No	4.114 (1.388-12.195)	**0.011**		1.963 (0.552-6.983)	0.298	
Yes	1.303 (1.120-1.515)	**<0.001**		1.939 (1.203-3.127)	**0.007**	
**Anti-platelet drugs**			0.384			0.999
No	1.390 (1.135-1.702)	**0.001**		1.290 (0.742-2.243)	0.367	
Yes	1.397 (1.122-1.740)	**0.003**		2.169 (1.049-4.488)	**0.037**	
**Hypoglycemic drugs**			0.406			0.521
No	1.829 (1.163-2.875)	**0.009**		0.811 (0.325-2.027)	0.655	
Yes	1.337 (1.144-1.564)	**<0.001**		1.679 (1.051-2.680)	**0.030**	
**Lipid-modifying drugs**			0.637			0.803
No	1.465 (1.173-1.828)	**<0.001**		1.553 (0.904-2.670)	0.111	
Yes	1.322 (1.081-1.618)	**0.007**		1.626 (0.778-3.397)	0.196	

Bold values indicate statistical significance (P < 0.05).

In both datasets, elevated WATyG correlated with higher mortality risk in clinically meaningful subgroups. In MIMIC-IV, continuous WATyG showed significant positive associations in patients aged ≥65 years (OR 1.817, 9%CI 1.449-2.279, P for trend<0.001) and those with CRMS (OR 1.420, 95%CI 1.183-1.705, P for trend<0.001), while WATyG quartiles revealed the highest risk in Q4 relative to Q1 in these subgroups (both P for trend<0.001). The private dataset demonstrated significant associations for continuous WATyG in males (P for trend =0.002) and patients with stroke (P for trend<0.001).

These results highlight WATyG’s robustness as a mortality predictor in patients with hypertension and KF. Consistent associations across CRMS and DKD subgroups, paired with meaningful trends in key populations (e.g., older patients, males), support its clinical relevance, even with subtle interaction effects for age and respiratory failure, the overall positive link between WATyG and mortality risk remains stable.

## 4 Discussions

The present study offers novel insights into the dynamic trajectories of the TyG index and its association with all-cause hospital mortality among high-risk patients with hypertension and KF using data from two datasets. Through longitudinal cluster analysis, we identified four distinct TyG trajectory patterns: rapidly increasing, rapidly decreasing, persistent high and stable low, each exhibiting differential prognostic implications. Our findings underscore that dynamic changes of TyG significantly link to mortality risk, reinforcing the TyG index as a robust metabolic marker for risk stratification in this vulnerable population.

### 4.1 Prognostic mechanisms of TyG trajectory patterns

The differential prognostic implications of TyG trajectories may be attributed to underlying molecular alterations in triglyceride metabolism. Cluster 1 (rapidly increasing TyG) likely reflects progressive dysregulation of LPL-mediated triglyceride clearance or elevated ANGPTL3/4/8 expression, promoting atherogenic lipoprotein accumulation and endothelial dysfunction (20, 30). In contrast, Cluster 2 (rapidly decreasing TyG) may indicate effective response to glucose/lipid-lowering medications, consistent with its high lipid-modifying and hypoglycemic drug use, and improved LPL activity, reducing cardiorenal lipotoxicity. Cluster 3 (persistent high TyG) may suggest sustained insulin resistance and impaired ApoC-II/A-V function (19), even with substantial hypoglycemic drug use, which offsets clinical intervention effects, which may be related to drug target resistance (e.g., decreased insulin receptor sensitivity) or comorbid uncontrolled inflammatory factors. Cluster 4 (stable low TyG) may be characterized by intact triglyceride metabolism, preserved LPL clearance, balanced ANGPTL3/4/8 regulation, and lower burdens of insulin resistance, DKD and CRMS; based on data from two cohorts, moderate lipid-modifying drug use and higher ACEI/ARB adoption may further mitigate atherogenic stress and underpin this cluster’s superior prognosis. Specifically, ACEI/ARB can inhibit the renin-angiotensin system, reduce the expression of ANGPTL3 (31), thereby enhancing LPL-mediated triglyceride clearance and maintaining metabolic homeostasis.

### 4.2 Comparison with prior work

The association between dynamic TyG trajectories and clinical outcomes has been a key research focus. Some longitudinal studies (32–37) have demonstrated that elevated TyG levels predict poor prognosis. Shi et al. (35) reported that high-fluctuation TyG was correlated with increased mortality in patients with atrial fibrillation. Beyond its link to mortality, accumulating evidence has indicated that TyG trajectories are also associated with the risk of incident diseases (38, 39), which further supports the role of TyG as a robust prognostic biomarker. Cai et al. (38) described a nonlinear relationship between TyG levels and AKI risk in patients with acute myocardial infarction, showing that the highest TyG quartile was associated with a 2.14-fold higher risk compared with the lowest quartile. These observations align with the present study’s finding that persistent high TyG levels in hospitalized patients reflect the severity of underlying insulin resistance, thereby providing empirical support for the hypothesis that therapeutic interventions targeting TyG reduction may mitigate disease progression and improve clinical outcomes. Notably, this study extends these findings to acute care settings, demonstrating that even short-term TyG elevation during hospitalization can adversely affect patient prognosis. Unlike previous research that primarily analyzed baseline TyG values (15, 21, 25, 40, 41), our longitudinal trajectory modeling captures dynamic temporal metabolic shifts, a factor critical for prognostic assessment. To our knowledge, this preliminary study is among the first to explore associations between dynamic TyG index trajectories and all-cause hospital mortality in patients with hypertension and KF.

Further, some studies have predominantly focused on long-term TyG trajectories over several years to explore associations with chronic disease progression or population-level outcomes (32–34, 42, 43). Lu et al. (32) analyzed 4,700 patients with CRMS and demonstrated that elevated cumulative TyG levels, calculated as mean TyG × time from 2011 to 2015, were linearly associated with a 13% increased stroke risk, with persistent high-TyG clusters showing the highest mortality. While these studies yield critical insights into chronic disease mechanisms, they primarily analyze longitudinal TyG changes in outpatient or general populations over extended periods. In contrast, the present analysis focuses on short-term TyG fluctuations during hospitalization, a high-risk clinical window characterized by rapid metabolic perturbations, to demonstrate that even transient metabolic dysregulation predicts in-hospital mortality. This acute inpatient setting differs from prior outpatient-focused research, underscoring the need for context-specific metabolic monitoring to optimize real-time risk stratification.

Additionally, while some investigators have explored composite indices combining the TyG index with anthropometric measures, including body mass index (BMI) or waist circumference (33, 34), our analysis focuses specifically on TyG trajectories. Anthropometric parameters including waist circumference and BMI exhibit limited short-term variability during hospitalization, rendering them suboptimal for acute prognostic assessment. We thus prioritized TyG trajectory analysis as a core metabolic marker, given its unique ability to reflect dynamic metabolic stress in acute care settings. This approach aligns with Ning et al. (37), who identified increasing and decreasing TyG trajectory phenotypes within the first 72 hours post-admission in sepsis patients, reported that an increasing trajectory correlated with significantly higher 28-day mortality. Such phenotypic heterogeneity underscores the clinical utility of trajectory analysis for identifying high-risk subgroups, particularly in acute care settings.

### 4.3 Novel TyG metrics and prognostic utility

A key strength of our study lies in the introduction of the time-weighted cumulative TyG metric (WATyG), which quantifies metabolic exposure during hospitalization. We observed 50.5% and 148.3% increased mortality risk in the highest WATyG quartile (Q4) compared to the lowest WATyG quartile (Q1) in the MIMIC-IV and private dataset, respectively, with RCS analysis showing a linear relationship in both datasets. These results concur with Cheng et al.’s (36) findings, where the TyG variability ratio (TyGVR) was calculated as (average TyG - baseline TyG)/baseline TyG during hospitalization, and showed linear associations with both in-hospital and 1-year mortality in ICU patients. While TyGVR focused on variability (change from baseline relative to baseline), our WATyG captured time-weighted cumulative exposure. Despite these methodological differences, both studies demonstrated that dynamic TyG assessment (via TyGVR or WATyG) enhances risk prediction.

Besides, we conducted multivariate analyses that were adjusted for treatment - related covariates (mainly drugs), including anti - hypertensive, anti - platelet, hypoglycemic and lipid - modifying drugs. The persistent significance of TyG trajectories and WATyG after medication adjustment confirms their independent prognostic value, as these drugs typically target triglyceride metabolism or insulin resistance. This key finding indicates that TyG dynamics may reflect intrinsic metabolic derangements rather than secondary effects of pharmacotherapy, thereby supporting the clinical utility of TyG trajectories for risk stratification.

In this study, age, respiratory failure, electrolyte disturbance, and lipid-modifying drug use modified the TyG-mortality association in patients with hypertension and KF, while TyG indices showed stable prognostic value across CRMS subgroups. The stronger association in elderly patients may reflect age-related metabolic dysregulation and visceral adiposity-induced inflammation (17, 44, 45), whereas attenuated associations in those with respiratory failure or electrolyte disturbance could be attributed to concurrent organ dysfunction (46, 47). Notably, TyG indices maintained reliable utility across most comorbidities and showed differential associations by lipid-modifying, anti-hypertensive, and anti-platelet drug use, capturing medication-related metabolic burden. These findings support TyG-based risk stratification and personalized interventions in high-risk subgroups.

The TyG trajectories identified in this study carry significant implications for clinical practice and healthcare policy. Integrating TyG into electronic health records could enable real-time risk alert systems, flagging patients with upward trajectories for early intervention. For example, rapid TyG elevation may prompt intensified glycemic and lipid control or adjustment of renin-angiotensin system inhibitors, potentially mitigating cardiorenal deterioration (15, 44). Notably, the survival benefit observed in Cluster 2 (rapid TyG decline) underscores the clinical value of metabolic interventions to improve endothelial function and reduce inflammation (48, 49). At the public health level, our findings advocate for updating chronic disease management guidelines to emphasize dynamic metabolic assessment. Traditional single-timepoint metrics often fail to capture the progressive nature of insulin resistance in hypertension-CKD comorbidity, whereas TyG trajectories provide a longitudinal lens for risk stratification (44, 45). Integrating TyG into predictive models alongside established biomarkers may enhance precision in resource allocation for high-risk patients, ultimately reducing hospitalizations and healthcare costs (29, 32–34, 39). By integrating mechanistic insights and actionable clinical tools, TyG trajectories offer a paradigm shift from reactive to proactive metabolic management in cardiorenal care.

### 4.4 Limitations and future directions

While our findings support a link between metabolic fluctuations and adverse outcomes in this high-risk population, the observational study design precludes causal inference, and several limitations must be noted. First, the retrospective design limits causal conclusions, and despite multivariate adjustments and multicenter data, residual biases from unmeasured factors (e.g., diet, physical activity, medication adherence) may persist. Second, excluding patients with short hospital stays (<48 hours) or fewer than two TyG measurements introduces selection bias. The excluded patients may suffer from severe acute conditions such as acute cardiorenal events or refractory shock, or have undergone brief hospitalizations. These patients are prone to rapid TyG elevations driven by acute inflammation or organ dysfunction and likely represent a distinct high-mortality subgroup. Their exclusion means this high-risk stratum was not captured in trajectory analyses potentially underestimating mortality risk associated with extreme TyG fluctuations and limiting generalizability to critically ill or short-stay patients. Third, the lack of post-discharge follow-up restricts outcomes to in-hospital mortality, narrowing generalizability to acute care settings. Fourth, clustering analysis relied only on baseline and final TyG measurements, omitting intermediate fluctuations that may reflect clinically relevant changes. Future studies should adopt standardized protocols (e.g., daily TyG monitoring) to capture both macro-trends and micro-fluctuations. Fifth, the novel WATyG metric has not been validated against gold-standard measures of insulin resistance such as the euglycemic clamp. Larger, rigorously designed cohorts are needed to validate these associations and elucidate mechanisms.

## 5 Conclusions

In this multicenter study of high-risk patients with hypertension and KF, we identified four distinct dynamic TyG trajectories (rapidly increasing, rapidly decreasing, persistent high and stable low), with rapidly increasing and persistent high patterns significantly associated with elevated hospital mortality. The time-weighted average TyG (WATyG) metric emerged as a robust prognostic marker, underscoring the clinical significance of cumulative metabolic exposure in predicting outcomes. These findings highlight the value of integrating dynamic TyG monitoring into risk stratification strategies, particularly for early risk stratification. While our observational design limits causal inference, the multicenter cohort enhances generalizability. Future prospective studies are warranted to validate causal relationships and explore whether targeted interventions modifying elevated TyG trajectories could improve survival in this vulnerable population.

## Data Availability

The original contributions presented in the study are included in the article/**Supplementary Material**. Further inquiries can be directed to the corresponding author/s.

## Supplementary material

Supplementary Figure 1 Restricted cubic spline (RCS) analysis of WATyG-mortality association across four TyG trajectory clusters in patients with hypertension and kidney failure in the MIMIC-IV dataset.

Supplementary Figure 2 Restricted cubic spline (RCS) analysis of WATyG-mortality association across four TyG trajectory clusters in patients with hypertension and kidney failure in the private dataset.

Supplementary Table 1 Subgroup analysis of the association between TyG trajectories and mortality risks in patients with hypertension and kidney failure in the private dataset.

Supplementary Table 2 Subgroup analysis of the association between WATyG quartiles and mortality in patients with hypertension and kidney failure in the MIMIC-IV dataset.

Supplementary Table 3 Subgroup analysis of the association between WATyG quartiles and mortality risks in patients with hypertension and kidney failure in the private dataset.

## References

[1] Zannad F McGuire DK Ortiz A . Treatment strategies to reduce cardiovascular risk in persons with chronic kidney disease and Type 2 diabetes. J Internal Med. (2025) 297:460–78. doi: 10.1111/joim.20050, PMID: 39739537 PMC12033002

[2] Jager KJ Kovesdy C Langham R Rosenberg M Jha V Zoccali C . A single number for advocacy and communication-worldwide more than 850 million individuals have kidney diseases. Kidney Int. (2019) 96:1048–50. doi: 10.1016/j.kint.2019.07.012, PMID: 31582227

[3] G.C.K.D. Collaboration . Global, regional, and national burden of chronic kidney disease, 1990-2017: a systematic analysis for the Global Burden of Disease Study 2017. Lancet (London England). (2020) 395:709–33. doi: 10.1016/s0140-6736(20)30045-3, PMID: 32061315 PMC7049905

[4] Hamrahian SM Falkner B . Hypertension in chronic kidney disease. Adv Exp Med Biol. (2017) 956:307–25. doi: 10.1007/5584_2016_84, PMID: 27873228

[5] Muntner P Anderson A Charleston J Chen Z Ford V Makos G . Hypertension awareness, treatment, and control in adults with CKD: results from the Chronic Renal Insufficiency Cohort (CRIC) Study. Am J Kidney Dis. (2010) 55:441–51. doi: 10.1053/j.ajkd.2009.09.014, PMID: 19962808 PMC2866514

[6] Chen TK Knicely DH Grams ME . Chronic kidney disease diagnosis and management: A review. Jama. (2019) 322:1294–304. doi: 10.1001/jama.2019.14745, PMID: 31573641 PMC7015670

[7] Marassi M Fadini GP . The cardio-renal-metabolic connection: a review of the evidence. Cardiovasc diabetol. (2023) 22:195. doi: 10.1186/s12933-023-01937-x, PMID: 37525273 PMC10391899

[8] Thomas MC Brownlee M Susztak K Sharma K Jandeleit-Dahm KA Zoungas S . Diabetic kidney disease. Nat Rev Dis primers. (2015) 1:15018. doi: 10.1038/nrdp.2015.18, PMID: 27188921 PMC7724636

[9] Zhang Y Chu C Zhong Z Luo YB Ning FF Guo N . High triglyceride-glucose index is associated with poor cardiovascular outcomes in Chinese acute coronary syndrome patients without diabetes mellitus who underwent emergency percutaneous coronary intervention with drug-eluting stents. Front Endocrinol. (2023) 14:1101952. doi: 10.3389/fendo.2023.1101952, PMID: 36875470 PMC9975349

[10] Simental-Mendía LE Hernández-Ronquillo G Gamboa-Gómez CI Gómez-Díaz R Rodríguez-Morán M Guerrero-Romero F . The triglycerides and glucose index is associated with elevated blood pressure in apparently healthy children and adolescents. Eur J pediatrics. (2019) 178:1069–74. doi: 10.1007/s00431-019-03392-x, PMID: 31081518

[11] Gao Q Lin Y Xu R Luo F Chen R Li P . Positive association of triglyceride-glucose index with new-onset hypertension among adults: a national cohort study in China. Cardiovasc diabetol. (2023) 22:58. doi: 10.1186/s12933-023-01795-7, PMID: 36927705 PMC10022268

[12] Kunutsor SK Seidu S Kurl S Kurl S Laukkanen JA . Baseline and usual triglyceride-glucose index and the risk of chronic kidney disease: a prospective cohort study. GeroScience. (2024) 46:3035–46. doi: 10.1007/s11357-023-01044-5, PMID: 38180700 PMC11009217

[13] Liao Y Zhang R Shi S Zhao Y He Y Liao L . Triglyceride-glucose index linked to all-cause mortality in critically ill patients: a cohort of 3026 patients. Cardiovasc diabetol. (2022) 21:128. doi: 10.1186/s12933-022-01563-z, PMID: 35804386 PMC9270811

[14] Lee SH Park SY Choi CS . Insulin resistance: from mechanisms to therapeutic strategies. Diabetes Metab J. (2022) 46:15–37. doi: 10.4093/dmj.2021.0280, PMID: 34965646 PMC8831809

[15] Ye Z An S Gao Y Xie E Zhao X Guo Z . Association between the triglyceride glucose index and in-hospital and 1-year mortality in patients with chronic kidney disease and coronary artery disease in the intensive care unit. Cardiovasc diabetol. (2023) 22:110. doi: 10.1186/s12933-023-01843-2, PMID: 37179310 PMC10183125

[16] Wang M Teng T Zhang N Xu J Dong Z Jiao Q . Derivatives of the triglyceride-glucose index and their association with incident hypertension in prehypertensive individuals: a 4-year cohort study augmented by mendelian randomization. Cardiovasc diabetol. (2025) 24:284. doi: 10.1186/s12933-025-02813-6, PMID: 40646540 PMC12247458

[17] Tchernof A Després JP . Pathophysiology of human visceral obesity: an update. Physiol Rev. (2013) 93:359–404. doi: 10.1152/physrev.00033.2011, PMID: 23303913

[18] Gaudet D Karwatowska-Prokopczuk E Baum SJ Hurh E Kingsbury J Bartlett VJ . Vupanorsen, an N-acetyl galactosamine-conjugated antisense drug to ANGPTL3 mRNA, lowers triglycerides and atherogenic lipoproteins in patients with diabetes, hepatic steatosis, and hypertriglyceridaemia. Eur Heart J. (2020) 41:3936–45. doi: 10.1093/eurheartj/ehaa689, PMID: 32860031 PMC7750927

[19] Wolska A Dunbar RL Freeman LA Ueda M Amar MJ Sviridov DO . Apolipoprotein C-II: New findings related to genetics, biochemistry, and role in triglyceride metabolism. Atherosclerosis. (2017) 267:49–60. doi: 10.1016/j.atherosclerosis.2017.10.025, PMID: 29100061 PMC5705268

[20] Scicchitano P Amati F Ciccone MM D'Ascenzi F Imbalzano E Liga R . Hypertriglyceridemia: molecular and genetic landscapes. Int J Mol Sci. (2024) 25:6364. doi: 10.3390/ijms25126364, PMID: 38928071 PMC11203941

[21] Zhang R Shi S Chen W Wang Y Lin X Zhao Y . Independent effects of the triglyceride-glucose index on all-cause mortality in critically ill patients with coronary heart disease: analysis of the MIMIC-III database. Cardiovasc diabetol. (2023) 22:10. doi: 10.1186/s12933-023-01737-3, PMID: 36639637 PMC9838037

[22] Yang Z Gong H Kan F Ji N . Association between the triglyceride glucose (TyG) index and the risk of acute kidney injury in critically ill patients with heart failure: analysis of the MIMIC-IV database. Cardiovasc diabetol. (2023) 22:232. doi: 10.1186/s12933-023-01971-9, PMID: 37653418 PMC10472684

[23] Lukito AA Kamarullah W Huang I Pranata R . Association between triglyceride-glucose index and hypertension: A systematic review and meta-analysis. Narra J. (2024) 4:e951. doi: 10.52225/narra.v4i2.951, PMID: 39280320 PMC11394170

[24] Dakota I Huang W Wijayanto MA Nurhafizah A Khairunnisa AR Rachmayanti S . Prognostic value of triglyceride-glucose index on predicting major adverse cardiovascular events in hypertensive patients: a systematic review and meta-analysis. Am J Prev Cardiol. (2025) 22:100996. doi: 10.1016/j.ajpc.2025.100996, PMID: 40290417 PMC12032867

[25] Lv L Xiong J Huang Y He T Zhao J . Association between the triglyceride glucose index and all-cause mortality in critically ill patients with acute kidney injury. Kidney Dis (Basel Switzerland). (2024) 10:69–78. doi: 10.1159/000535891, PMID: 38322625 PMC10843181

[26] Zhang Y Li G Li J Jian B Wang K Chen J . The triglyceride-glucose index and acute kidney injury risk in critically ill patients with coronary artery disease. Renal failure. (2025) 47:2466818. doi: 10.1080/0886022x.2025.2466818, PMID: 39972619 PMC11843639

[27] Cui H Liu Q Wu Y Cao L . Cumulative triglyceride-glucose index is a risk for CVD: a prospective cohort study. Cardiovasc diabetol. (2022) 21:22. doi: 10.1186/s12933-022-01456-1, PMID: 35144621 PMC8830002

[28] Feng M Zhou J . Relationship between time-weighted average glucose and mortality in critically ill patients: a retrospective analysis of the MIMIC-IV database. Sci Rep. (2024) 14:4721. doi: 10.1038/s41598-024-55504-9, PMID: 38413682 PMC10899565

[29] Huo RR Zhai L Liao Q You XM . Changes in the triglyceride glucose-body mass index estimate the risk of stroke in middle-aged and older Chinese adults: a nationwide prospective cohort study. Cardiovasc diabetol. (2023) 22:254. doi: 10.1186/s12933-023-01983-5, PMID: 37716947 PMC10505325

[30] Zhang R . The ANGPTL3-4–8 model, a molecular mechanism for triglyceride trafficking. Open Biol. (2016) 6:150272. doi: 10.1098/rsob.150272, PMID: 27053679 PMC4852456

[31] Umanath K Lewis JB . Update on diabetic nephropathy: core curriculum 2018. Am J Kidney Dis. (2018) 71:884–95. doi: 10.1053/j.ajkd.2017.10.026, PMID: 29398179

[32] Lu L Chen Y Liu B Li X Wang J Nie Z . Association between cumulative changes of the triglyceride glucose index and incidence of stroke in a population with cardiovascular-kidney-metabolic syndrome stage 0-3: a nationwide prospective cohort study. Cardiovasc diabetol. (2025) 24:202. doi: 10.1186/s12933-025-02754-0, PMID: 40355933 PMC12070779

[33] Wu Y Yang Y Zhang J Liu S Zhuang W . The change of triglyceride-glucose index may predict incidence of stroke in the general population over 45 years old. Cardiovasc diabetol. (2023) 22:132. doi: 10.1186/s12933-023-01870-z, PMID: 37296457 PMC10257314

[34] Zhu X Xu W Song T Wang X Wang Q Li J . Changes in the combination of the triglyceride-glucose index and obesity indicators estimate the risk of cardiovascular disease. Cardiovasc diabetol. (2024) 23:192. doi: 10.1186/s12933-024-02281-4, PMID: 38844974 PMC11157789

[35] Shi S Xue F Jiang T Ling L . Association between trajectory of triglyceride-glucose index and all-cause mortality in critically ill patients with atrial fibrillation: a retrospective cohort study. Cardiovasc diabetol. (2025) 24:278. doi: 10.1186/s12933-025-02838-x, PMID: 40640797 PMC12243274

[36] Cheng L Zhang F Xue W Yu P Wang X Wang H . Association of dynamic change of triglyceride-glucose index during hospital stay with all-cause mortality in critically ill patients: a retrospective cohort study from MIMIC IV2.0. Cardiovasc Diabetol. (2023) 22:142. doi: 10.1186/s12933-023-01874-9, PMID: 37330498 PMC10276426

[37] Ning YL Xu XH Niu XL Zhang Y Zhou JH Sun C . The triglyceride-glucose index dynamic trajectory reveals the association between the clinical subphenotype of insulin resistance and mortality in patients with sepsis. BMC Infect diseases. (2024) 24:1083. doi: 10.1186/s12879-024-10005-y, PMID: 39354398 PMC11443761

[38] Cai D Xiao T Chen Q Gu Q Wang Y Ji Y . Association between triglyceride glucose and acute kidney injury in patients with acute myocardial infarction: a propensity score−matched analysis. BMC Cardiovasc Disord. (2024) 24:216. doi: 10.1186/s12872-024-03864-5, PMID: 38643093 PMC11031878

[39] Yuan Y Chen S Lin C Huang X Lin S Huang F . Association of triglyceride-glucose index trajectory and frailty in urban older residents: evidence from the 10-year follow-up in a cohort study. Cardiovasc diabetol. (2023) 22:264. doi: 10.1186/s12933-023-02002-3, PMID: 37775740 PMC10542691

[40] Ma M Hao J Yu K Lv Y Liu X Liu F . Association between triglyceride glucose index and all-cause mortality in patients with critical atrial fibrillation in the MIMIC-IV database. Sci Rep. (2025) 15:13484. doi: 10.1038/s41598-025-96735-8, PMID: 40251213 PMC12008299

[41] Sharifi-Zahabi E Nasiri N Hajizadeh-Sharafabad F Sharifi M Saber A . Triglyceride-glucose index and the risk of in-hospital and ICU all-cause mortality: a systematic review and meta-analysis of observational studies. Nutr diabetes. (2025) 15:8. doi: 10.1038/s41387-025-00366-x, PMID: 39987150 PMC11846995

[42] Zhang M Guan Q Guo Z Guan C Jin X Dong H . Changes in the triglyceride-glucose-body mass index estimate the risk of hypertension among the middle-aged and older population: a prospective nationwide cohort study in China in the framework of predictive, preventive, and personalized medicine. EPMA J. (2024) 15:611–27. doi: 10.1007/s13167-024-00380-6, PMID: 39635021 PMC11612070

[43] Wang A Tian X Zuo Y Chen S Meng X Wu S . Change in triglyceride-glucose index predicts the risk of cardiovascular disease in the general population: a prospective cohort study. Cardiovasc diabetol. (2021) 20:113. doi: 10.1186/s12933-021-01305-7, PMID: 34039351 PMC8157734

[44] Tao S Yu L Li J Huang L Huang X Zhang W . Association between the triglyceride-glucose index and 1-year major adverse cardiovascular events in patients with coronary heart disease and hypertension. Cardiovasc diabetol. (2023) 22:305. doi: 10.1186/s12933-023-02018-9, PMID: 37940943 PMC10633928

[45] Lei L Liang H Qu Y Zhong Q Zhang Q Dai L . Association between triglyceride-glucose index and worsening renal function in the elderly. Front Nutr. (2022) 9:951564. doi: 10.3389/fnut.2022.951564, PMID: 36505264 PMC9730025

[46] Sarnak MJ Amann K Bangalore S Cavalcante JL Charytan DM Craig JC . Chronic kidney disease and coronary artery disease: JACC state-of-the-art review. J Am Coll Cardiol. (2019) 74:1823–38. doi: 10.1016/j.jacc.2019.08.1017, PMID: 31582143

[47] Yildirim F Yildiz AB Kanbay M . A promising tool: triglyceride-glucose index to stratify the risk of cardiovascular events in chronic kidney disease. Clin Kidney J. (2022) 15:1653–6. doi: 10.1093/ckj/sfac084, PMID: 36003667 PMC9394708

[48] Erqou S Adler AI Challa AA Fonarow GC Echouffo-Tcheugui JB . Insulin resistance and incident heart failure: a meta-analysis. Eur J Heart failure. (2022) 24:1139–41. doi: 10.1002/ejhf.2531, PMID: 35502564 PMC9262840

[49] Tsuda A Ishimura E Uedono H Ochi A Nakatani S Morioka T . Association of albuminuria with intraglomerular hydrostatic pressure and insulin resistance in subjects with impaired fasting glucose and/or impaired glucose tolerance. Diabetes Care. (2018) 41:2414–20. doi: 10.2337/dc18-0718, PMID: 30217931

