# Supplementary material for: Dynamic trajectories of the triglyceride-glucose index link to all-cause hospital mortality in patients with hypertension and kidney failure: a multicenter study
Source: Front Endocrinol (Lausanne). 2026 Jan 9;16:1698418. doi: 10.3389/fendo.2025.1698418 (PMC12827127; doi:10.3389/fendo.2025.1698418)
Supplement: Supplementary Figure 1 — Restricted cubic spline (RCS) analysis of WATyG-mortality association across four TyG trajectory clusters in patients with hypertension and kidney failure in the MIMIC-IV dataset. [file DataSheet1.docx]

Supplementary Material


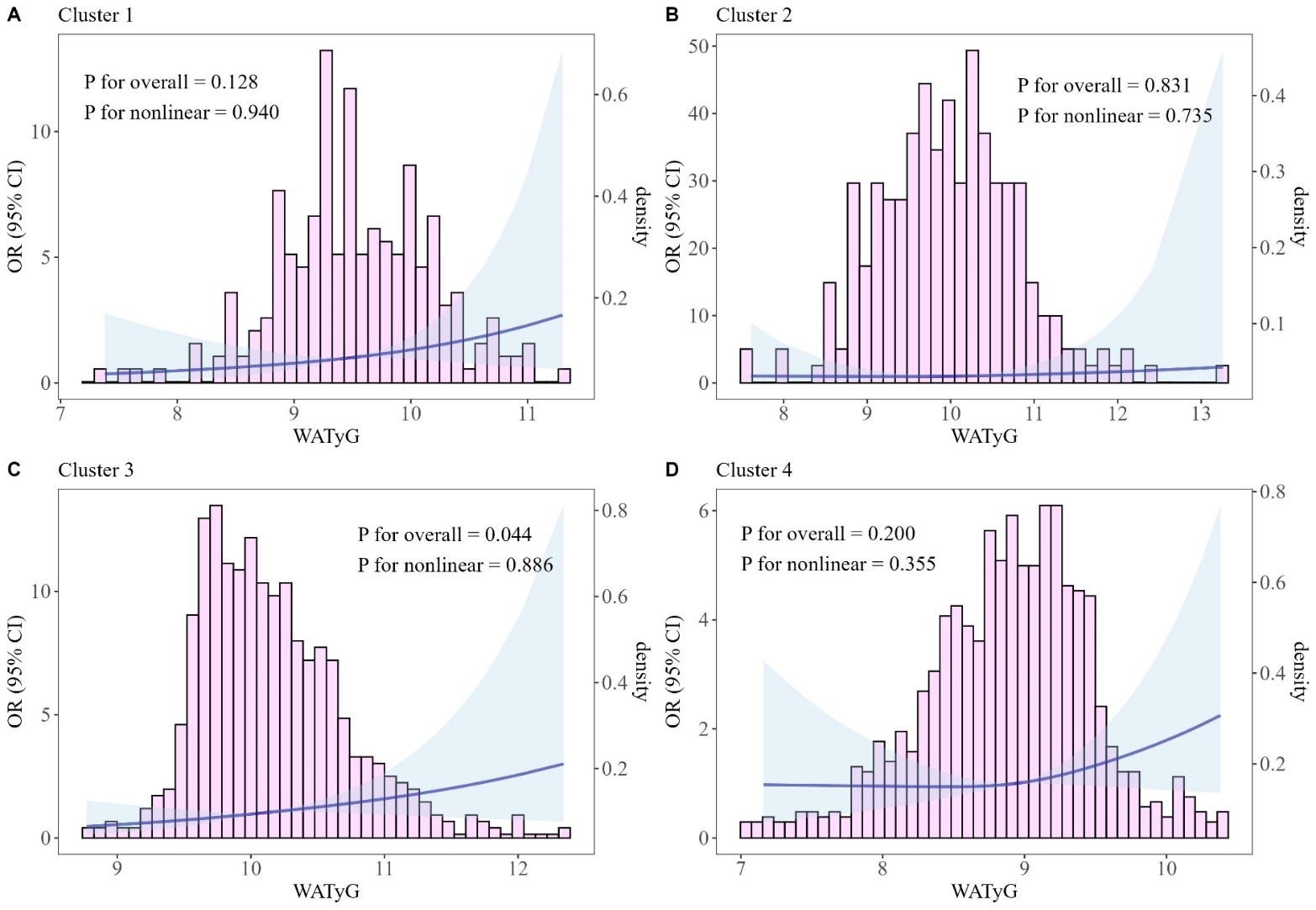


# Figure S1. Restricted cubic spline (RCS) analysis of WATyG-mortality association across four TyG trajectory clusters in patients with hypertension and kidney failure in the MIMIC-IV dataset.


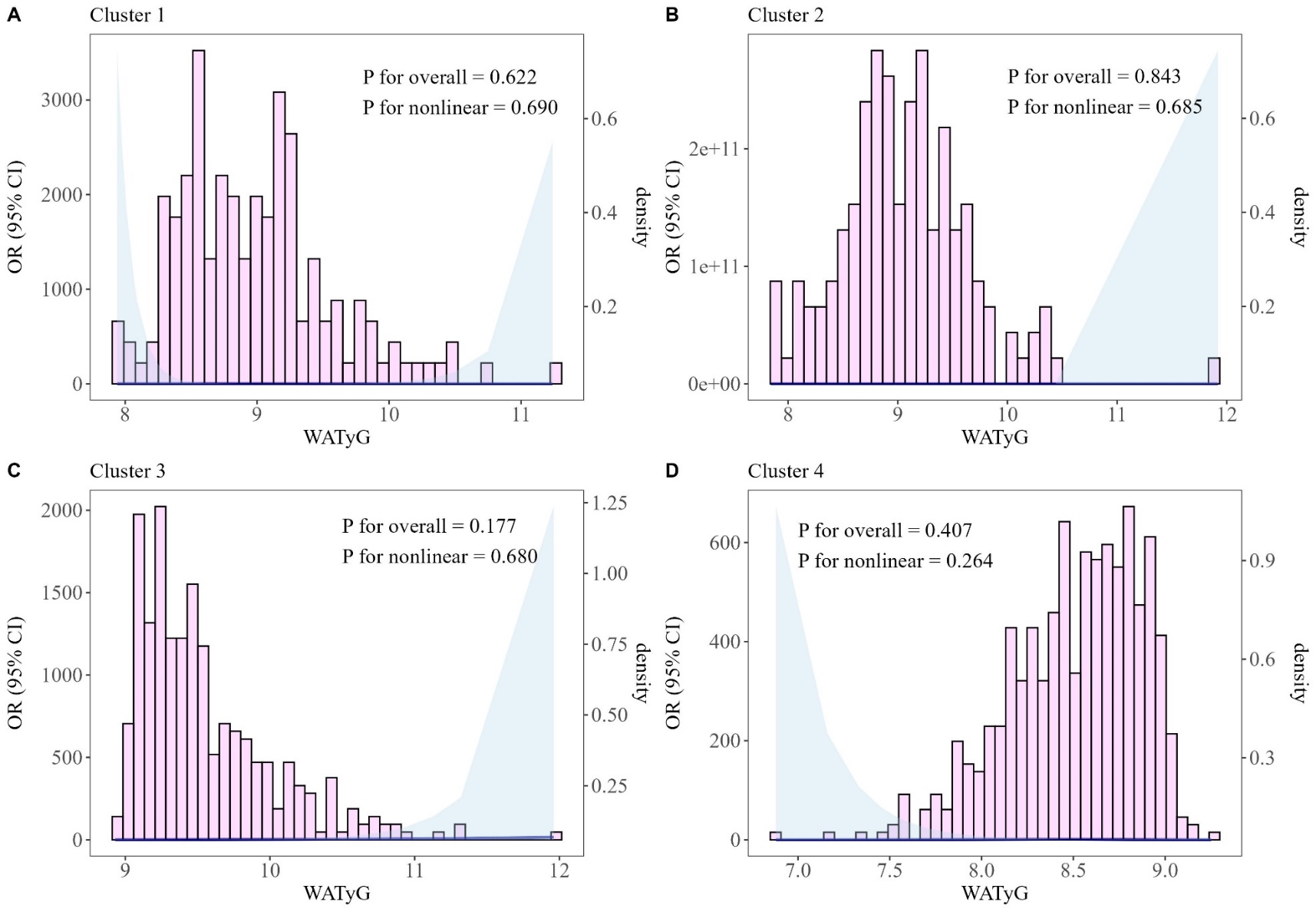


# Figure S2. Restricted cubic spline (RCS) analysis of WATyG-mortality association across four TyG trajectory clusters in patients with hypertension and kidney failure in the private dataset.

# Table S1. Subgroup analysis of the association between TyG trajectories and mortality risks in patients with hypertension and kidney failure in the private dataset.

| **Variable** | **Cluster 1** | **Cluster 2** | **Cluster 3** | **Cluster 4** | **P for Trend** | **P for interaction** |
| --- | --- | --- | --- | --- | --- | --- |
| **Age** |  |  |  |  |  | **0.024** |
| <65 | Reference | 0.000 (0.000-Inf) | 0.000 (0.000-Inf) | 0.000 (0.000-Inf) | 0.992 |  |
| ≥65 | Reference | 0.149 (0.038-0.579) | 0.583 (0.242-1.402) | 0.406 (0.181-0.909) | 0.089 |  |
| **Gender** |  |  |  |  |  | 0.567 |
| Female | Reference | 0.008 (0.000-0.220) | 0.048 (0.006-0.373) | 0.109 (0.016-0.754) | 0.06 |  |
| Male | Reference | 0.477 (0.121-1.884) | 0.730 (0.281-1.901) | 0.286 (0.110-0.743) | **0.021** |  |
| **Cardio-renal-metabolic syndrome** |  |  |  |  |  | 0.686 |
| No | Reference | 0.195 (0.042-0.901) | 0.583 (0.196-1.739) | 0.224 (0.083-0.604) | **0.009** |  |
| Yes | Reference | 0.111 (0.015-0.806) | 0.329 (0.095-1.132) | 0.211 (0.054-0.821) | 0.063 |  |
| **Diabetic kidney disease** |  |  |  |  |  | 0.608 |
| No | Reference | 0.179 (0.057-0.565) | 0.534 (0.243-1.175) | 0.325 (0.150-0.701) | **0.023** |  |
| Yes | Reference | 40.738 (0.000-Inf) | 0.000 (0.000-Inf) | 0.000 (0.000-Inf) | 1 |  |
| **Hyperlipidemia** |  |  |  |  |  | 0.501 |
| No | Reference | 0.163 (0.051-0.524) | 0.526 (0.248-1.112) | 0.304 (0.144-0.640) | **0.01** |  |
| Yes | Reference | 0.005 (0.000-Inf) | 0.000 (0.000-Inf) | 1.985 (0.000-Inf) | 1 |  |
| **Ischaemic heart disease** |  |  |  |  |  | 0.121 |
| No | Reference | 0.182 (0.039-0.861) | 0.239 (0.070-0.819) | 0.152 (0.047-0.491) | **0.002** |  |
| Yes | Reference | 0.257 (0.044-1.516) | 0.762 (0.235-2.471) | 0.307 (0.091-1.032) | 0.126 |  |
| **Peripheral vascular disease** |  |  |  |  |  | 0.344 |
| No | Reference | 0.202 (0.060-0.677) | 0.457 (0.208-1.006) | 0.245 (0.108-0.552) | **0.002** |  |
| Yes | Reference | 0.000 (0.000-Inf) | Inf (0.000-Inf) | Inf(0.000-Inf) | 1 |  |
| **Respiratory failure** |  |  |  |  |  | 0.069 |
| No | Reference | 0.121 (0.029-0.508) | 0.560 (0.232-1.350) | 0.159 (0.059-0.423) | **0.002** |  |
| Yes | Reference | 4.256 (0.119-152.391) | 0.095 (0.007-1.275) | 0.803 (0.099-6.491) | 0.633 |  |
| **Heart failure** |  |  |  |  |  | 0.434 |
| No | Reference | 0.206 (0.035-1.216) | 0.253 (0.070-0.909) | 0.269 (0.079-0.913) | **0.03** |  |
| Yes | Reference | 0.196 (0.038-0.999) | 0.718 (0.230-2.240) | 0.296 (0.099-0.882) | 0.096 |  |
| **Arrhythmia** |  |  |  |  |  | 0.616 |
| No | Reference | 0.171 (0.039-0.752) | 0.479 (0.172-1.331) | 0.377 (0.137-1.039) | 0.164 |  |
| Yes | Reference | 0.141 (0.015-1.297) | 0.464 (0.109-1.970) | 0.209 (0.055-0.788) | 0.047 |  |
| **Diabetes** |  |  |  |  |  | 0.597 |
| No | Reference | 0.228 (0.054-0.970) | 0.557 (0.192-1.614) | 0.223 (0.084-0.593) | **0.007** |  |
| Yes | Reference | 0.076 (0.008-0.748) | 0.351 (0.099-1.246) | 0.215 (0.050-0.919) | 0.078 |  |
| **Stroke** |  |  |  |  |  | 0.131 |
| No | Reference | 0.262 (0.057-1.192) | 0.636 (0.244-1.657) | 0.395 (0.156-1.002) | 0.098 |  |
| Yes | Reference | 0.011 (0.000-0.289) | 0.178 (0.019-1.629) | 0.038 (0.004-0.374) | **0.026** |  |
| **Electrolyte Disturbance** |  |  |  |  |  | 0.848 |
| No | Reference | 0.185 (0.034-1.018) | 0.604 (0.208-1.755) | 0.238 (0.081-0.695) | **0.024** |  |
| Yes | Reference | 0.123 (0.018-0.848) | 0.340 (0.068-1.690) | 0.298 (0.073-1.216) | 0.178 |  |
| **Cancer** |  |  |  |  |  | 0.6 |
| No | Reference | 0.209 (0.067-0.654) | 0.485 (0.225-1.047) | 0.290 (0.133-0.632) | **0.008** |  |
| Yes | Reference | 0.000 (0.000-Inf) | 0.000 (0.000-Inf) | 3.480 (0.000-Inf) | 1.00 |  |
| **Chronic obstructive pulmonary disease** |  |  |  |  |  | 0.445 |
| No | Reference | 0.113 (0.012-1.110) | 0.430 (0.103-1.794) | 0.218 (0.049-0.957) | 0.096 |  |
| Yes | Reference | 0.241 (0.054-1.066) | 0.447 (0.160-1.244) | 0.376 (0.143-0.987) | 0.071 |  |
| **Anemia** |  |  |  |  |  | 0.105 |
| No | Reference | 0.042 (0.006-0.295) | 0.556 (0.210-1.471) | 0.418 (0.162-1.077) | 0.271 |  |
| Yes | Reference | 0.342 (0.051-2.268) | 0.316 (0.060-1.653) | 0.088 (0.012-0.658) | **0.015** |  |
| **Anti-hypertensive drugs** |  |  |  |  |  | **0.03** |
| No | Reference | 0.000 (0.000-Inf) | 0.000 (0.000-Inf) | 0.000 (0.000-Inf) | 0.988 |  |
| Yes | Reference | 0.336 (0.091-1.241) | 0.972 (0.382-2.471) | 0.493 (0.193-1.257) | 0.317 |  |
| **Anti-platelet drugs** |  |  |  |  |  | 0.309 |
| No | Reference | 0.199 (0.045-0.881) | 0.394 (0.138-1.125) | 0.211 (0.071-0.628) | **0.011** |  |
| Yes | Reference | 0.099 (0.011-0.862) | 0.655 (0.170-2.519) | 0.389 (0.107-1.409) | 0.229 |  |
| **Hypoglycemic drugs** |  |  |  |  |  | 0.759 |
| No | Reference | 0.178 (0.016-1.937) | 0.088 (0.009-0.820) | 0.242 (0.057-1.026) | 0.064 |  |
| Yes | Reference | 0.145 (0.035-0.603) | 0.591 (0.233-1.503) | 0.258 (0.092-0.721) | 0.047 |  |
| **Lipid-modifying drugs** |  |  |  |  |  | 0.247 |
| No | Reference | 0.108 (0.023-0.515) | 0.398 (0.141-1.128) | 0.168 (0.058-0.488) | **0.005** |  |
| Yes | Reference | 0.151 (0.017-1.333) | 0.527 (0.119-2.328) | 0.329 (0.081-1.342) | 0.237 |  |

# Table S2. Subgroup analysis of the association between WATyG quartiles and mortality in patients with hypertension and kidney failure in the MIMIC-IV dataset.

| **Variable** | **Q1** | **Q2** | **Q3** | **Q4** | **P for Trend** | **P for interaction** |
| --- | --- | --- | --- | --- | --- | --- |
| **Age** |  |  |  |  |  | **0.003** |
| <65 | Reference | 1.270 (0.769-2.098) | 1.018 (0.615-1.685) | 1.728 (1.057-2.827) | **0.049** |  |
| ≥65 | Reference | 1.574 (1.040-2.381) | 1.974 (1.288-3.025) | 3.661 (2.255-5.945) | **<0.001** |  |
| **Gender** |  |  |  |  |  | 0.149 |
| Female | Reference | 1.512 (0.898-2.546) | 1.899 (1.118-3.225) | 2.697 (1.555-4.679) | **<0.001** |  |
| Male | Reference | 1.324 (0.889-1.972) | 1.214 (0.810-1.819) | 1.837 (1.216-2.773) | **0.009** |  |
| **Cardio-renal-metabolic syndrome** |  |  |  |  |  | 0.136 |
| No | Reference | 1.325 (0.823-2.135) | 0.892 (0.530-1.501) | 2.013 (1.161-3.491) | 0.092 |  |
| Yes | Reference | 1.473 (0.963-2.253) | 1.758 (1.162-2.661) | 2.192 (1.442-3.330) | **<0.001** |  |
| **Diabetic kidney disease** |  |  |  |  |  | 0.708 |
| No | Reference | 1.395 (0.992-1.962) | 1.322 (0.933-1.872) | 2.287 (1.590-3.288) | **<0.001** |  |
| Yes | Reference | 2.103 (0.888-4.982) | 2.201 (0.948-5.109) | 2.096 (0.913-4.813) | 0.126 |  |
| **Hyperlipidemia** |  |  |  |  |  | 0.069 |
| No | Reference | 1.482 (0.973-2.255) | 0.982 (0.629-1.534) | 2.008 (1.255-3.213) | 0.038 |  |
| Yes | Reference | 1.219 (0.749-1.984) | 2.012 (1.254-3.228) | 2.227 (1.391-3.567) | **<0.001** |  |
| **Ischaemic heart diseases** |  |  |  |  |  | 0.205 |
| No | Reference | 1.423 (0.946-2.140) | 1.210 (0.795-1.841) | 2.019 (1.327-3.071) | **0.004** |  |
| Yes | Reference | 1.324 (0.801-2.191) | 1.750 (1.052-2.912) | 2.361 (1.388-4.015) | **<0.001** |  |
| **Peripheral vascular disease** |  |  |  |  |  | 0.457 |
| No | Reference | 1.322 (0.883-1.978) | 1.592 (1.061-2.388) | 2.203 (1.460-3.324) | **<0.001** |  |
| Yes | Reference | 1.737 (1.038-2.909) | 1.122 (0.657-1.916) | 1.964 (1.137-3.394) | 0.066 |  |
| **Respiratory failure** |  |  |  |  |  | **<0.001** |
| No | Reference | 1.866 (1.068-3.259) | 1.714 (0.932-3.153) | 4.572 (2.572-8.126) | **<0.001** |  |
| Yes | Reference | 1.206 (0.808-1.799) | 1.214 (0.822-1.793) | 1.272 (0.840-1.927) | 0.283 |  |
| **Heart failure** |  |  |  |  |  | 0.835 |
| No | Reference | 1.460 (0.973-2.191) | 1.358 (0.901-2.046) | 2.253 (1.490-3.406) | **<0.001** |  |
| Yes | Reference | 1.274 (0.761-2.134) | 1.441 (0.857-2.424) | 1.947 (1.121-3.379) | **0.018** |  |
| **Arrhythmia** |  |  |  |  |  | 0.314 |
| No | Reference | 1.433 (0.871-2.358) | 1.184 (0.722-1.944) | 1.816 (1.110-2.969) | **0.041** |  |
| Yes | Reference | 1.294 (0.859-1.948) | 1.431 (0.938-2.183) | 2.385 (1.519-3.744) | **<0.001** |  |
| **Diabetes** |  |  |  |  |  | 0.127 |
| No | Reference | 1.459 (0.982-2.169) | 1.431 (0.944-2.170) | 3.016 (1.916-4.749) | **<0.001** |  |
| Yes | Reference | 1.290 (0.759-2.193) | 1.302 (0.781-2.169) | 1.586 (0.963-2.613) | 0.081 |  |
| **Stroke** |  |  |  |  |  | 0.514 |
| No | Reference | 1.421 (0.997-2.025) | 1.460 (1.024-2.084) | 2.168 (1.505-3.123) | **<0.001** |  |
| Yes | Reference | 1.560 (0.744-3.272) | 1.750 (0.785-3.899) | 2.384 (1.033-5.501) | 0.045 |  |
| **Electrolyte Disturbance** |  |  |  |  |  | 0.101 |
| No | Reference | 2.015 (0.862-4.711) | 2.666 (1.094-6.494) | 2.754 (0.978-7.751) | 0.032 |  |
| Yes | Reference | 1.288 (0.913-1.818) | 1.186 (0.842-1.671) | 1.903 (1.346-2.691) | **<0.001** |  |
| **Cancer** |  |  |  |  |  | **0.006** |
| No | Reference | 1.262 (0.881-1.809) | 1.142 (0.795-1.642) | 1.626 (1.122-2.355) | **0.023** |  |
| Yes | Reference | 1.802 (0.874-3.714) | 2.153 (1.036-4.477) | 5.292 (2.397-11.681) | **<0.001** |  |
| **Chronic obstructive pulmonary disease** |  |  |  |  |  | **0.017** |
| No | Reference | 1.204 (0.740-1.958) | 0.886 (0.525-1.494) | 1.272 (0.747-2.164) | 0.63 |  |
| Yes | Reference | 1.555 (1.020-2.372) | 1.687 (1.109-2.567) | 2.606 (1.694-4.007) | **<0.001** |  |
| **Anemia** |  |  |  |  |  | 0.170 |
| No | Reference | 1.118 (0.606-2.062) | 1.495 (0.827-2.701) | 2.524 (1.365-4.669) | **0.002** |  |
| Yes | Reference | 1.504 (1.037-2.180) | 1.289 (0.880-1.888) | 1.942 (1.313-2.872) | **0.004** |  |
| **Anti-hypertensive drugs** |  |  |  |  |  | **0.008** |
| No | Reference | 5.425 (0.703-41.870) | 5.103 (0.463-56.274) | 12.718 (1.475-109.631) | 0.025 |  |
| Yes | Reference | 1.366 (0.989-1.887) | 1.277 (0.922-1.769) | 1.902 (1.360-2.661) | **<0.001** |  |
| **Anti-platelet drugs** |  |  |  |  |  | 0.297 |
| No | Reference | 1.544 (1.000-2.383) | 1.501 (0.966-2.332) | 2.105 (1.339-3.310) | **0.003** |  |
| Yes | Reference | 1.237 (0.777-1.971) | 1.217 (0.761-1.947) | 2.257 (1.394-3.652) | **0.002** |  |
| **Hypoglycemic drugs** |  |  |  |  |  | 0.305 |
| No | Reference | 2.352 (1.128-4.903) | 1.189 (0.473-2.991) | 6.452 (2.278-18.275) | **0.005** |  |
| Yes | Reference | 1.235 (0.864-1.764) | 1.318 (0.929-1.868) | 1.877 (1.320-2.669) | **<0.001** |  |
| **Lipid-modifying drugs** |  |  |  |  |  | 0.609 |
| No | Reference | 1.493 (0.956-2.332) | 1.297 (0.821-2.049) | 2.127 (1.314-3.443) | **0.007** |  |
| Yes | Reference | 1.279 (0.816-2.004) | 1.546 (0.984-2.428) | 2.152 (1.362-3.399) | **<0.001** |  |

# Table S3. Subgroup analysis of the association between WATyG quartiles and mortality risks in patients with hypertension and kidney failure in the private dataset.

| **Variable** | **Q1** | **Q2** | **Q3** | **Q4** | **P for Trend** | **P for interaction** |
| --- | --- | --- | --- | --- | --- | --- |
| **Age** |  |  |  |  |  | 0.613 |
| <65 | Reference | 0.000 (0.000-Inf) | 0.000 (0.000-Inf) | 207150672802521088.000 (0.000-Inf) | 0.993 |  |
| ≥65 | Reference | 1.809 (0.713-4.590) | 1.144 (0.415-3.149) | 1.654 (0.606-4.512) | **0.572** |  |
| **Gender** |  |  |  |  |  | **0.054** |
| Female | Reference | 0.338 (0.045-2.558) | 0.176 (0.019-1.606) | 0.061 (0.006-0.616) | **0.016** |  |
| Male | Reference | 2.207 (0.702-6.938) | 1.119 (0.330-3.795) | 2.986 (0.985-9.058) | **0.116** |  |
| **Cardio-renal-metabolic syndrome** |  |  |  |  |  | 0.729 |
| No | Reference | 1.935 (0.641-5.843) | 1.800 (0.546-5.935) | 1.832 (0.575-5.834) | **0.367** |  |
| Yes | Reference | 1.254 (0.275-5.716) | 0.574 (0.109-3.019) | 1.471 (0.337-6.409) | 0.707 |  |
| **Diabetic kidney disease** |  |  |  |  |  | 0.748 |
| No | Reference | 1.926 (0.795-4.663) | 1.157 (0.443-3.023) | 1.689 (0.694-4.110) | **0.492** |  |
| Yes | Reference | 0.000 (0.000-Inf) | 58.520 (0.000-Inf) | 2853079.060 (0.000-Inf) | 1 |  |
| **Hyperlipidemia** |  |  |  |  |  | 0.142 |
| No | Reference | 1.973 (0.811-4.799) | 1.285 (0.501-3.298) | 1.915 (0.794-4.623) | **0.31** |  |
| Yes | Reference | 0.004 (0.000-Inf) | 0.000 (0.000-Inf) | 0.000 (0.000-Inf) | 1 |  |
| **Ischaemic heart diseases** |  |  |  |  |  | 0.659 |
| No | Reference | 2.931 (0.729-11.773) | 0.571 (0.115-2.831) | 1.671 (0.444-6.279) | 0.97 |  |
| Yes | Reference | 0.764 (0.210-2.773) | 1.125 (0.293-4.321) | 1.722 (0.437-6.787) | 0.298 |  |
| **Peripheral vascular disease** |  |  |  |  |  | 0.952 |
| No | Reference | 2.360 (0.905-6.153) | 1.675 (0.624-4.496) | 1.993 (0.775-5.127) | **0.305** |  |
| Yes | Reference | 0.000 (0.000-Inf) | 0.000 (0.000-Inf) | 0.000 (0.000-Inf) | 1 |  |
| **Respiratory failure** |  |  |  |  |  | **0.003** |
| No | Reference | 1.959 (0.609-6.304) | 1.996 (0.603-6.602) | 3.803 (1.213-11.929) | **0.023** |  |
| Yes | Reference | 7.554 (0.154-371.050) | 0.002 (0.000-1.348) | 0.000 (0.000-0.550) | 0.01 |  |
| **Heart failure** |  |  |  |  |  | 0.678 |
| No | Reference | 2.613 (0.646-10.571) | 0.836 (0.155-4.519) | 1.571 (0.383-6.446) | 0.964 |  |
| Yes | Reference | 1.533 (0.443-5.306) | 1.474 (0.401-5.421) | 2.429 (0.670-8.800) | 0.202 |  |
| **Arrhythmia** |  |  |  |  |  | 0.644 |
| No | Reference | 2.824 (0.842-9.472) | 0.823 (0.211-3.211) | 1.867 (0.591-5.896) | 0.68 |  |
| Yes | Reference | 1.208 (0.258-5.671) | 2.182 (0.438-10.870) | 1.356 (0.224-8.205) | 0.538 |  |
| **Diabetes** |  |  |  |  |  | 0.745 |
| No | Reference | 2.089 (0.710-6.141) | 1.830 (0.568-5.895) | 1.890 (0.611-5.851) | **0.353** |  |
| Yes | Reference | 1.258 (0.229-6.906) | 0.595 (0.102-3.479) | 1.569 (0.326-7.545) | 0.64 |  |
| **Stroke** |  |  |  |  |  | 0.387 |
| No | Reference | 1.866 (0.652-5.344) | 1.057 (0.333-3.357) | 1.346 (0.443-4.090) | **0.932** |  |
| Yes | Reference | 1.021 (0.110-9.522) | 0.358 (0.027-4.665) | 2.885 (0.340-24.459) | 0.244 |  |
| **Electrolyte Disturbance** |  |  |  |  |  | 0.483 |
| No | Reference | 2.144 (0.613-7.497) | 1.990 (0.498-7.955) | 2.875 (0.816-10.128) | **0.137** |  |
| Yes | Reference | 1.510 (0.291-7.838) | 0.636 (0.116-3.489) | 0.952 (0.192-4.727) | 0.715 |  |
| **Cancer** |  |  |  |  |  | 0.703 |
| No | Reference | 1.835 (0.737-4.566) | 1.152 (0.431-3.081) | 1.780 (0.714-4.438) | 0.405 |  |
| Yes | Reference | 0.099 (0.000-Inf) | 237.115 (0.000-Inf) | 0.000 (0.000-Inf) | 1 |  |
| **Chronic obstructive pulmonary disease** |  |  |  |  |  | 0.517 |
| No | Reference | 2.690 (0.449-16.110) | 2.604 (0.360-18.826) | 2.021 (0.314-13.022) | 0.665 |  |
| Yes | Reference | 1.793 (0.604-5.324) | 0.851 (0.259-2.795) | 1.275 (0.401-4.059) | 0.946 |  |
| **Anemia** |  |  |  |  |  | 0.087 |
| No | Reference | 1.290 (0.449-3.710) | 0.677 (0.200-2.296) | 0.936 (0.327-2.677) | 0.655 |  |
| Yes | Reference | 3.053 (0.310-30.041) | 4.510 (0.509-39.923) | 5.901 (0.696-50.045) | 0.101 |  |
| **Anti-hypertensive drugs** |  |  |  |  |  | 0.083 |
| No | Reference | 23.603 (1.149-484.892) | 0.356 (0.003-50.360) | 3.570 (0.127-100.721) | 0.622 |  |
| Yes | Reference | 1.670 (0.526-5.303) | 1.899 (0.615-5.870) | 2.498 (0.840-7.432) | **0.102** |  |
| **Anti-platelet drugs** |  |  |  |  |  | 0.690 |
| No | Reference | 1.504 (0.489-4.630) | 0.636 (0.174-2.327) | 0.865 (0.277-2.706) | 0.547 |  |
| Yes | Reference | 3.692 (0.598-22.808) | 3.120 (0.476-20.438) | 6.227 (0.983-39.460) | **0.076** |  |
| **Hypoglycemic drugs** |  |  |  |  |  | 0.276 |
| No | Reference | 3.125 (0.675-14.477) | 0.678 (0.080-5.717) | 0.922 (0.136-6.271) | 0.709 |  |
| Yes | Reference | 1.259 (0.366-4.328) | 1.423 (0.420-4.819) | 1.757 (0.559-5.520) | **0.296** |  |
| **Lipid-modifying drugs** |  |  |  |  |  | 0.845 |
| No | Reference | 1.992 (0.644-6.159) | 1.122 (0.318-3.964) | 1.727 (0.563-5.294) | 0.553 |  |
| Yes | Reference | 1.398 (0.263-7.444) | 1.243 (0.210-7.355) | 2.374 (0.408-13.800) | **0.329** |  |
